# Semiconducting Single‐Wall Antimony Oxide Nanotubes and Oxidotritelluridoantimonate(V) Anions

**DOI:** 10.1002/chem.71106

**Published:** 2026-05-11

**Authors:** Huanyu He, Oleg Janson, Vitaliy Romaka, Michael Ruck

**Affiliations:** ^1^ Faculty of Chemistry and Food Chemistry TUD Dresden University of Technology Dresden Germany; ^2^ Leibniz Institute for Solid State and Materials Research Dresden Dresden Germany

**Keywords:** antimony(V), cetineites, hydrothermal synthesis, nanotubes, semiconductors

## Abstract

Black, needle‐shaped crystals of K_6_[Sb_12_O_18_](SbOTe_3_)_2_·*n*H_2_O (*n* ≈ 5) were grown in high yield from an alkaline medium under hydrothermal conditions. Microporous, single‐walled [Sb_12_O_18_] nanotubes dominate the hexagonal crystal structure of the cetineite‐type. Antimony(III) atoms form the outer and oxygen atoms the inner wall of the nanotube, making it radially dipolar, similar to a cylindrical capacitor. The inner wall is decorated with potassium cations, and water molecules fill the remaining internal volume of the nanotube. Stacks of the hitherto unknown oxidotritelluridoantimonate(V) anion, (SbOTe_3_)^3−^, fill the trigonal channels in the packing of the nanotubes. As with other modifications of Sb_2_O_3_, the filled bonding and empty antibonding states of the nanotubes are separated by a wide band gap of about 3.6 eV. In this energy gap, the molecular orbitals of the (SbOTe_3_)^3−^ anions are located. Electronic excitation involves a charge transfer from the anions to the nanotubes, which are thereby heavily n‐doped and exhibit semiconducting behavior. Modification of the reaction conditions results in the crystallization of the tellurium‐rich cetineite K_6_[Sb_12_O_18_](Te_3_)_2_(Te_2_)_3_·5H_2_O or the cetineite K_18_[Sb_36_O_54_](SbOTe_3_)_2_(Te_2_)_6_·22H_2_O, which contains [Sb_36_O_54_] nanotubes with an even wider diameter.

## Graphical abstract

Antimony(V) binds directly to tellurium(−II) in the (SbOTe_3_)^3−^ anion. The latter has been found between Sb_2_O_3_ nanotubes in a microporous cetineite structure. The Sb_2_O_3_ nanotube is positively polarized on its outer surface and negatively polarized on its inner surface, corresponding to a cylindrical capacitor. Substantial charge transfer from the anions to the nanotubes results in pseudo‐one‐dimensional semiconducting behavior.

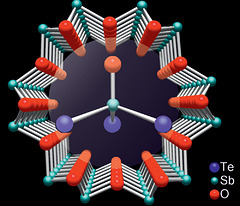

## 1 Introduction

In the research for advanced functional materials for applications in energy conversion, storage, and catalysis, the correlation between structure and property remains a significant theme. A focal point of current investigations is the rational design of porous semiconducting materials, which are of key importance in the development of next‐generation materials for photocatalysis, gas separation, and molecular sensing. One‐dimensional (1D) semiconductors, such as nanotubes and nanowires, with small band gaps offer excellent charge mobility and a high surface area [1], whereas microporous materials, such as metal–organic frameworks (MOFs), covalent organic frameworks (COFs) [2], and zeolites [3], provide ordered channels and size‐selective adsorption, which are essential for sorption‐based or diffusion‐limited processes.

Crystalline microporous semiconductors have a great potential for application, and materials with structures analogous to the minerals of the cetineite group emerge as a promising subclass [4, 5]. Such cetineite‐type structures were successfully synthesized with the hydrothermal approach. K_3_Sb_7_O_9_Se_3_·3H_2_O = K_6_(H_2_O)_6_[Sb_12_O_18_][SbSe_3_]_2_, as the first synthetic crystalline microporous cetineite material, was identified as a photo‐semiconductor with a band gap of 2.06 eV [6]. Significantly narrower band gaps of 0.25 and 0.85 eV were found in the tellurium‐containing microporous cetineites K_18_(H_2_O)_18_[Sb_36_O_54_][Te_36_] and Rb_18_[Sb_36_O_54_](SbTe_3_)_2_(Te_2_)_6_ [7, 8]. Despite their promising structures, microporous semiconductors of the cetineite‐type are still relatively unexplored. In this study, we present new representatives of this interesting class of compounds, which are obtained in high yields within a few hours by hydrothermal reactions using optimized synthesis protocols. The newly synthesized compounds contain an additional chemical rarity, the oxidotritelluridoantimonate(V) anion (SbOTe_3_)^3–^, which exhibits a direct chemical bond between antimony in its maximum positive oxidation state (a strong oxidizing agent) and tellurium in its maximum negative oxidation state.

## 2 Results and Discussion

### 2.1 Crystal Structure

**FIGURE 1:**
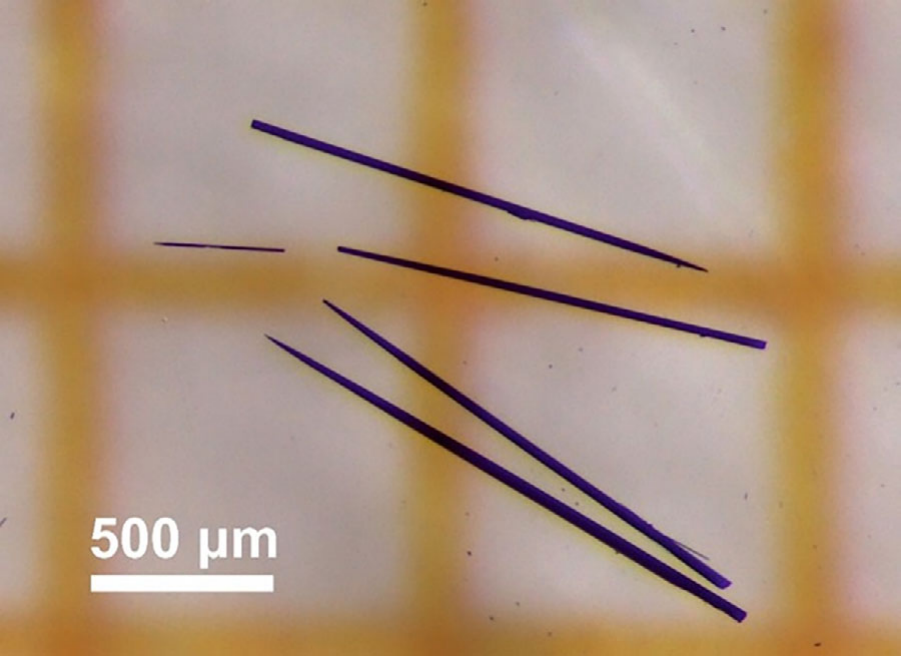
Photograph of crystals of cetineite **1**.

**FIGURE 2:**
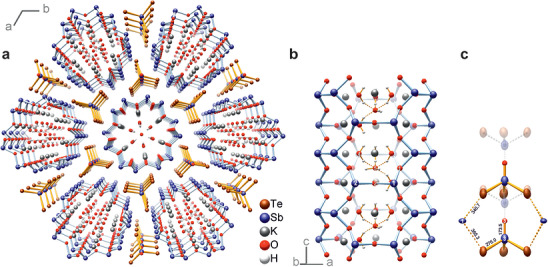
(a) Hexagonal crystal structure of **1**. The [Sb_12_O_18_] nanotubes are filled with hydrated potassium cations; trigonal (SbOTe_3_)^3−^ pyramids fill the channels between the nanotubes. (b) [Sb_12_O_18_] nanotube in the crystal structure of **1**. The (3,3) armchair nanotube is filled with clusters of five water molecules and potassium cations. The figure shows an arbitrary arrangement of the disordered water molecules that is consistent with the refined occupancies. (c) Trigonal pyramids (SbOTe_3_)^3−^ stacked along the *c*‐axis in the crystal structure of **1**. The polar molecules must have the same orientation within one stack. The orientational disorder (transparently drawn atoms) is probably due to the lack of order between different stacks. Some of the secondary bonds Te···Sb1 are indicated. Interatomic distances are given in pm. The ellipsoids enclose a space in which, with 99%, probability the electron density of the atoms can be found at 100 K.

The new cetineite K_6_[Sb^III^_12_O_18_](Sb^V^OTe_3_)_2_·*n*H_2_O (*n* ≈ 5) (**1**) was crystallized with a yield of 94% from an alkaline medium (Figures 1, S2 and S3 and Tables S1 and S2). X‐ray diffraction on a black, needle‐shaped single‐crystal (Figure S4, Table S4–S8) revealed a hexagonal structure in the space group *P*6_3_/*m* with the lattice parameters *a* = 1523.92(5) pm and *c* = 562.21(2) pm at 100(1) K (Figure 2a). The characteristic building blocks of the structure are single‐wall [Sb_12_O_18_] nanotubes, which form a hexagonal packing parallel to the *c*‐axis (Figure 2b). Antimony(III) atoms form the outer, oxygen atoms the inner wall of the nanotube, making the intrinsically uncharged nanotube radially dipolar, similar to a cylindrical capacitor. The [Sb_12_O_18_] nanotube is a form of Sb_2_O_3_ that is not found as a binary modification. Nonetheless, as in cubic α‐Sb_2_O_3_, which consists of Sb_4_O_6_ molecules (Sb─O 197.84(6) pm) [9], and in orthorhombic β‐Sb_2_O_3_, which is composed of chains (Sb─O 198.35(9)–202.07(9) pm) [10], the antimony(III) atoms are bonded to three μ‐bridging oxygen atoms (Sb─O 196.8(2)–201.5(3) pm). With this connectivity, crown‐shaped [Sb_6_O_6_] rings are formed and arranged analogous to a (3,3) armchair carbon nanotube. Bond angles O─Sb─O ranging from 87.6(1)° to 91.7(1)°, suggest bonding via the Sb‐5*p* orbitals. The inner diameter of the nanotube is characterized by O···O distances of about 940 pm. The [Sb_12_O_18_] network exhibits *D*_6h_ pseudosymmetry (rod group *p*6_3_/*mmc*), although the crystallographic symmetry is reduced to *C*_6h_ due to the packing.

The inner wall is decorated with potassium cations that are coordinated by six oxygen atoms of the [Sb_12_O_18_] network and three or four water molecules, which fill the interior of the nanotube. The hydrogen atoms (H4) of these coordinating water molecules (O4) point away from the potassium cations and thus toward the central axis of the nanotube. There, another water molecule (O5) is located. The position of the latter is occupied to 50%, that of the surrounding molecules to 67%. This avoids conflicts in the hydrogen bond system (short H4···H5 distances) and creates clusters of five water molecules (Figure 2b). The donor–acceptor distance O4···O5 is 239 pm, and the O4–H4···O5 angle is 150°. X‐ray diffraction analysis of several crystals revealed a variation in the water content, with a range of four to five molecules per formula unit.

Spatially separated from the potassium cation by the wall of the [Sb_12_O_18_] nanotube, (SbOTe_3_)^3−^ anions fill the trigonal channels in the packing of the nanotubes (Figure 2a,c). The novel (SbOTe_3_)^3−^ anion has the shape of a trigonal pyramid with molecular *C*_3v_ symmetry (crystallographic *C*_3_). The interatomic distances only allow the same orientation of the polar molecules within one stack. The observed orientational disorder of the anions is not superimposed by the mirror planes of the space group *P*6_3_/*m*, but is probably due to a lack of ordering information between the stacks in the different channels, as shown by refinements in the polar space group *P*6_3_. This is a common phenomenon in cetineite‐type structures with (SbS_3_)^3−^, (SbSe_3_)^3−^, or (SbTe_3_)^3−^ groups [5, 8]. It is noteworthy that none of the previously reported cetineites have been found to contain antimony(V).

### 2.2 Chemical Bonding

The hitherto unknown oxidotritelluridoantimonate(V) anion, (SbOTe_3_)^3−^, is a remarkable species. Maximum positive and negative oxidation states on directly bonded heavy main group elements are not expected for two reasons. First, the stability of the maximum positive oxidation states decreases towards the higher periods, which is mainly due to the increasing stabilization of the *s*‐electrons by relativistic effects. Therefore, very strong oxidizing agents are needed. These are, for the same reason, not found among the heavy main group elements, except that they are themselves in the highest oxidation state. Second, heavy elements with extremely different oxidation states are expected to undergo a redox reaction with each other, resulting in a more balanced electronic situation due to their similar electronegativity. The Pauling electronegativity values of the neighboring elements antimony (2.0) and tellurium (2.1) differ only marginally, yet formally justify the assigned oxidation states. It is astonishing that tellurium can compete with oxygen as a bonding partner of the antimony(V) atom. Among the few examples for molecular heavy‐atom chalcogenopnictides with the strongly diverting oxidation states −II and +V are (AsSe_4_)^3−^ [11] and (SbS_4_)^3−^ [12]. Compared to (SbOTe_3_)^3–^, both have larger differences in the electronegativity of their constituting elements. It therefore seems appropriate to consider the electronic situation in this anion more comprehensively, based on chemical arguments and underpinned by quantum chemical calculations and bonding analyses.

The Sb3─Te distance in the (SbOTe_3_)^3−^ anion is 276.01(5) pm, and hence slightly longer than the bond lengths reported for (Sb^III^Te_3_)^3−^ (272.6 pm in Rb_18_(Sb_36_O_54_)_6_(SbTe_3_)_2_(Te_2_)_6_ (**3**) [5], 278.3 pm in Na_3_SbTe_3_ [13], and 278.7 pm in K_3_SbTe_3_ [14, 15]. The bond angle Te−Sb3−Te of 101.65(2)° is also very similar to those in Na_3_SbTe_3_ (100.08°) and K_3_SbTe_3_ (101.98°). Overall, the additional oxygen atoms appear to have only a little effect on the Sb─Te bonding, which had been analyzed earlier [13]. The Sb3─O3 bond length *d* = 173.51(9) pm (O3─Sb3─Te 116.47(2)°) is not characteristic of a specific oxidation state of Sb. The bond valence *s*, using the formula *s* = exp[(*R* − *d*)/37 pm] [16] with the bond valence parameter *R*_SbO_ = 194.2 pm [16], is calculated to *s*_SbO_ = 1.75. Together with the three Sb3─Te bonds (*s*_SbTe_ = 1.06; *R*_SbTe_ = 278 pm), a bond valence sum of 4.93 results, which confirms the assigned oxidation state of the Sb3 atom.

A density functional theory (DFT) based full‐relativistic relaxation of the crystal structure (in space group *P*1) was performed for **1**. Details can be found in the Supporting Information. The result confirmed the experimentally determined structure, with the exception of a different arrangement of the water molecules. More importantly, the calculations reproduced the shape of the (SbOTe_3_)^3−^ anion, yet with a longer Sb3─O distance (187.6±0.1 pm) and a wider Te─Sb3─Te angle (275±1 pm, 110±2°). However, the experimentally determined Sb─O distance and the Te─Sb─Te angle are less reliable than their standard deviations suggest, since both are affected by uncertainties arising from the orientational disorder of this group (cf. elongated ellipsoid of Sb3 in Figure 2c).

**FIGURE 3:**
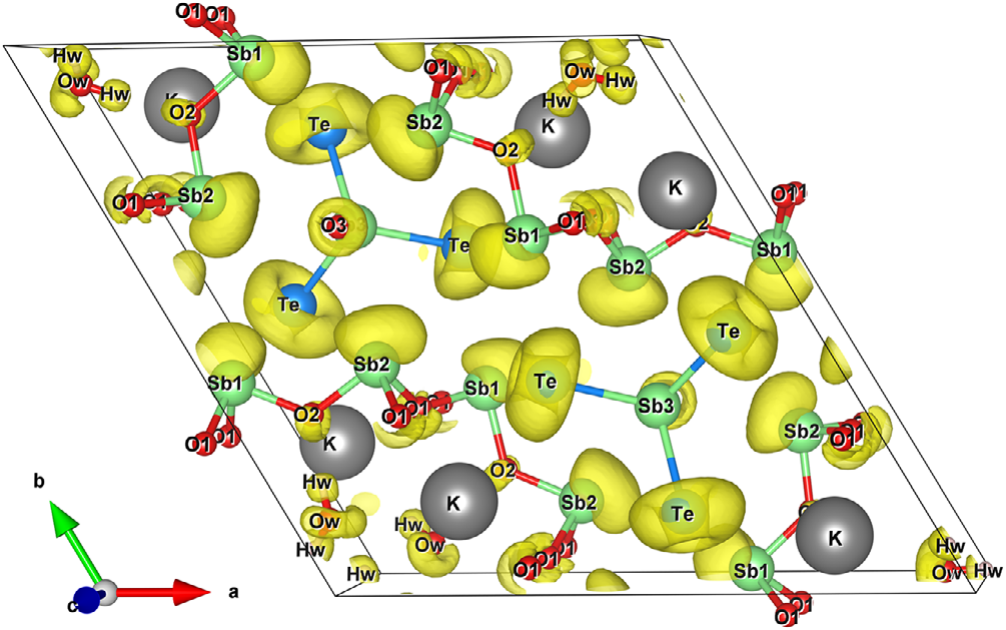
The isosurface of the electron localization function at ELF = 0.84 of the hydrated cetineite **1**.

The Integrated Crystal Orbital Hamilton Population (ICOHP) and the Integrated Crystal Orbital Bond Index (ICOBI) for the atomic pairs (Table S11) confirm a very strong Sb3─O3 bond within the (SbOTe_3_)^3−^ anion, in accordance with the short interatomic distance. The high –ICOHP value of 6.5 in combination with ICOBI = 1.0 suggests a robust covalent bond character. The three Sb3─Te bonds have single‐bond character (ICOBI = 0.813) but a weaker covalent contribution (–ICOHP = 3.8). The electron localization function (ELF) of **1** (Figure 3) reveals high values on the oxygen atoms of the [Sb_12_O_18_] nanotube and the water molecules, which aligns with the presence of two lone pairs at each oxygen atom. Similarly, the ELF maxima on the atoms Sb1 and Sb2 in the nanotubes correspond to the lone pair of an antimony atom in oxidation state +III.

In the (SbOTe_3_)^3−^ anion, the toroidal ELF maxima around Te and O atoms indicate their lone pairs, whose repulsive effect determines the pyramidal shape of the anion. Remarkably, there is no evidence for a (non‐spherical) lone pair on Sb3, suggesting that its valence electrons are largely involved in the chemical bonding, which supports the oxidation state +V. The density of states (DOS), however, shows that the 5*s* orbitals of all Sb atoms are filled and lie far below the Fermi level (*E*_F_), which contradicts a full oxidation of Sb3 in the (SbOTe_3_)^3−^ anion (Figures S7 and S8). The distribution of the atomic charges (Table S12) indicates that in the (SbOTe_3_)^3−^ anion, the O atoms are twice as negatively charged than the Te atoms. The positive charge of the Sb3 atom in the (SbOTe_3_)^3−^ anion is lower than that of the Sb1 and Sb2 atoms in the [Sb_12_O_18_] nanotube, although Sb3 has the higher (formal) oxidation state. If the calculated atomic charges are doubled and rounded to integer oxidation states, the resulting (Sb^II^O^−II^Te^−I^_3_)^3−^ approximates the actual charge distribution better than the formally correct oxidation states (Sb^V^O^−II^Te^−II^_3_)^3−^.

No special aspects were found for the other components. The positive charge on the K atom corresponds to a K^+^ cation, and the charges on the H and O atoms in the crystallization water are close to those in pure H_2_O.

### 2.3 Dehydration

A sample of cetineite **1** was heated at a rate of 5 K min^−1^ in a stream of argon inside a thermobalance (Figure S4). The crystals retained their internal water up to 400 °C. At this temperature, all water of crystallization is released in one step, resulting in a 2.6% mass loss (calculated 2.9% for five equivalents). This confirms the water content determined by structure refinement and shows that the water is strongly bonded within the microporous nanotube. Upon heating to 1200 °C, all tellurium (−24.6 wt.‐%), most of the potassium (−1.25 wt.‐%), and a fraction of the oxygen (−0.51 wt.‐%) and antimony (−3.9 wt.‐%) evaporate (Table S10) [17, 18].

Single‐crystal structure determinations at 298 K (25 °C), 323 K (50 °C), and 343 K (70 °C) showed no substantial changes compared to the structure at 100 K (structure data in Table S5). However, the unit cell at 343 K is significantly smaller (Δ*V* = −0.2%) than at 323 K (Table S4). This suggests a loss of water during the measurement in the continuous stream of warm and dry N_2_ gas that was used for heating. The water loss appears to be only a few percent, although the high standard deviations of the occupancy parameters of the water molecules do not allow for reliable quantification.

**FIGURE 4:**
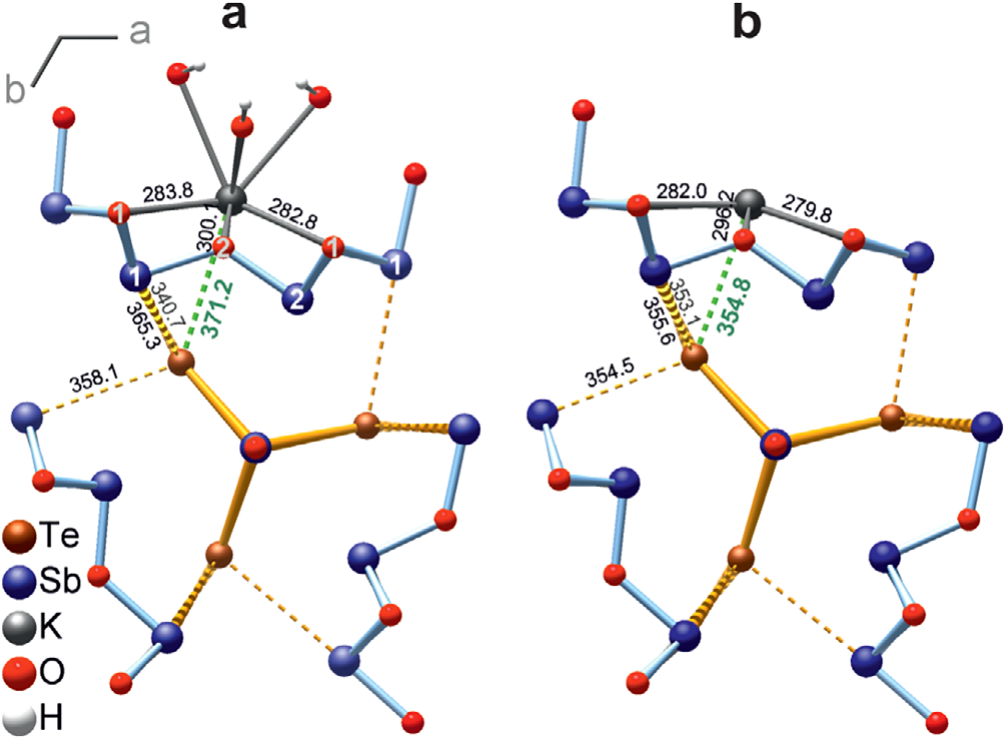
Local environment of the (SbOTe_3_)^3−^ anion and the potassium cation in (a) the crystal structure of **1** at 100 K and (b) the calculated structure of the dehydrated compound **1a**. Interatomic distances are given in pm.

A DFT‐based full‐relativistic relaxation of the crystal structure (in space group *P*1) for a water‐free variant of this cetineite structure (**1a**) revealed that even full dehydration should not have a strong effect on the crystal structure (Figure S7). The unit cell of **1a** expands relative to the optimized cell of **1** by Δ*V*/*V* = +0.6% with Δ*a*/*a* = +0.4% and Δ*c*/*c* = −0.4%. The K^+^ cation, which is no longer hydrated in **1a**, shortens its distances to the oxygen atoms of the nanotube by about 4 pm and its distance to the nearest tellurium atom of the (SbOTe_3_)^3−^ anion by about 16 pm (Figure 4). The enthalpy of hydration (Δ_hyd_ *H*) was calculated using Equation 1:

(1) $$\Delta_{\mathrm{hyd}}H=E_{\mathrm{tot}}\left(1\right)-E_{\mathrm{tot}}\left(1a\right)-5\times E_{\mathrm{tot}}\left(H_{2}O\right)$$

*E*_tot_ is the total energy of the optimized crystal structure of the hydrated cetineite **1**, the dehydrated cetineite **1a**, and the H_2_O molecule. The calculated molar enthalpy of hydration Δ_hyd,m_ *H* = −61.5 kJ mol(H_2_O)^−1^ can be compared with the standard hydration enthalpy for a potassium cation, which was experimentally determined to be −322(2) kJ mol^−1^ [19]. Given that approximately six water molecules reside in the first hydration shell of a potassium cation in aqueous solution [20], a hydration enthalpy of about −54 kJ mol(H_2_O)^−1^ is expected as a rough estimate. The molar enthalpy of evaporation for pure water is even lower (approximately 44 kJ mol^−1^ at room temperature). This is in line with the high temperature for evaporating water that was found in the TG experiment.

### 2.4 Electronic Structure and Electric Conductivity

**FIGURE 5:**
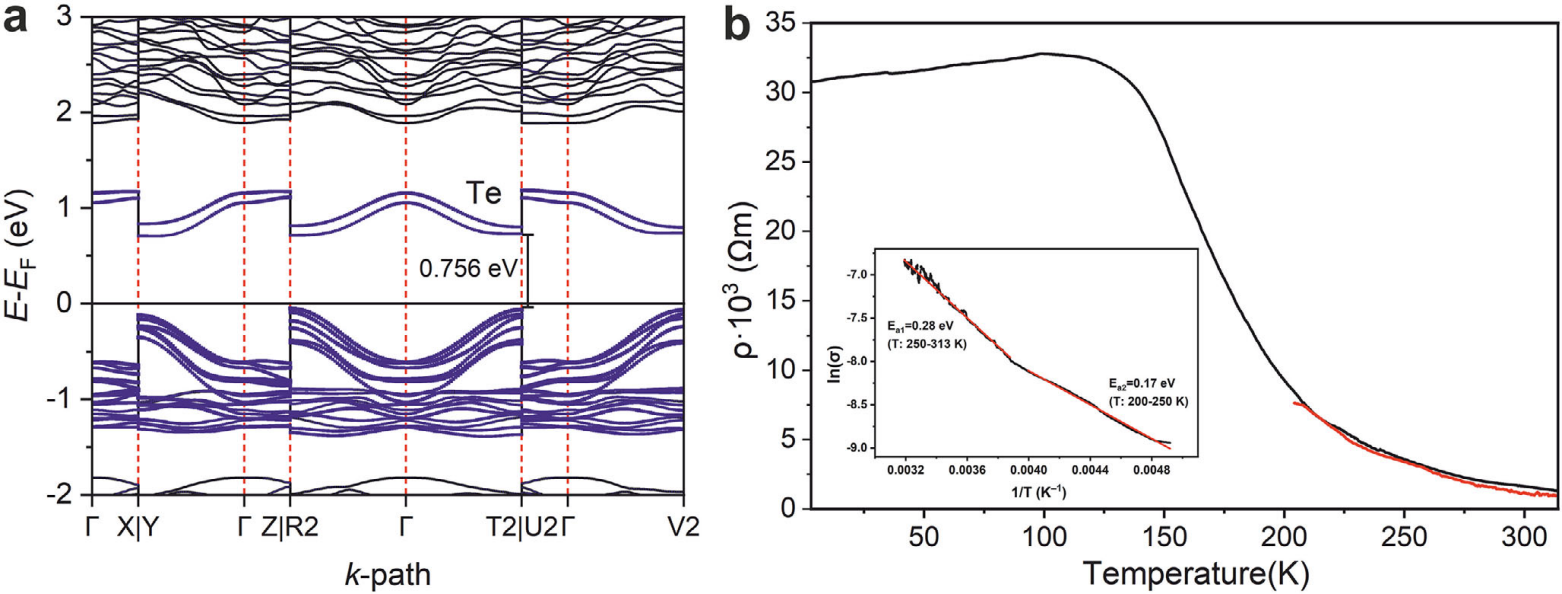
(a) Calculated band structure of the optimized structure of cetineite **1** showing a band gap of 0.756 eV. (b) Electrical resistivity of a crystal of **1** measured along the needle axis in a four‐probe setup (red line) and a two‐probe setup (black line; data rescaled to the four‐probe measurement).

The electronic band structures of the cetineite **1** (Figure 5a) and its dehydrated form **1a** (Figure S10) are almost identical. The water just adds localized states several eV below *E*_F_. Both electronic band structures show a wide gap of about 3.6 eV between the filled bonding and the empty antibonding Sb─O states. This corresponds to the gap of colorless α‐Sb_2_O_3_. The dark color of the needles of **1** originates from excitations of electrons located in the molecular states of the (SbOTe_3_)^3−^ anion. They lie within the gap of the [Sb_12_O_18_] nanotubes, and the anion's HOMO−LUMO gap defines *E*_F_. The calculated band gaps are 0.756 eV for **1** and 0.793 eV for **1a**. Inclusion of spin‐orbit coupling has no significant effect on the band structure. The band gaps are then only slightly smaller, with 0.754 eV for **1** and 0.788 eV for **1a**. Despite all calculated gaps being indirect, they almost match the corresponding direct (optical) gaps.

The states directly below *E*_F_ are Sb3─O3 and Sb3─Te bonding with strong contributions of the *p*‐orbitals of Te and O3 atoms. The empty states directly above *E*_F_ are the corresponding antibonding molecular orbitals. The (SbOTe_3_)^3−^ pyramid's ability to act as an antenna for electromagnetic radiation (e.g., light) is ensured by its axial polarity and the pronounced polarizability of the valence shells of the main group elements. Energies exceeding 2 eV (620 nm, orange colored light) also allow direct electronic excitation into the conduction band of the [Sb_12_O_18_] nanotubes and thus electronic transport along the needle axis. The holes, however, remain localized on the anions. This excitation involves electron hopping from a tellurium atom of the (SbOTe_3_)^3−^ anion to an antimony atom of the nanotube over at least 341 pm. Therefore, the excited state, which corresponds to an n‐doping of the nanotubes, is expected to have a long lifetime and to create a capacitance. In this context, the structure of the [Sb_12_O_18_] nanotube must be emphasized again, which produces a permanent radial dielectric polarization similar to that in a cylindrical capacitor. The spatial separation of K^+^ cations and (SbOTe_3_)^3–^ anions by the nanotubes enhances this effect.

These considerations are in line with the measured temperature‐dependence of the electrical resistivity along the needle axis of a crystal of cetineite **1** (Figures 5b and S6). It shows a high ohmic resistance, which increases exponentially on cooling. The specific resistance at room temperature is approximately 1 kΩ m, which is similar to that of undoped silicon. In this comparison, it should be noted that silicon is a 3D bulk and not a microporous 1D semiconductor. From the Arrhenius plot of the high‐temperature regime (inset of Figure 5b), a band gap between 0.17 and 0.28 eV is deduced. These values are significantly smaller than the calculated band gap. Rather, they correspond to the small energy gaps between the lowest conduction bands of the nanotubes. Below 140 K, the resistance becomes nearly independent of temperature and exhibits a slight positive temperature gradient. Such behavior is known for semimetals and heavily n‐doped semiconductors [21]. These observations can be explained by considering that only the nanotubes provide a continuous conduction path. Through charge transfer from the (SbOTe_3_)^3−^ anions, the nanotubes acquire a modest number of electrons in the antibonding states that comprise their conduction bands (at approximately +2 eV). The flat dispersion of these bands and their low charge carrier density match the measured high resistivity and semiconducting behavior below 140 K.

The insertion of electronic states associated with the (SbOTe_3_)^3−^ anions into the wide band gap of the [Sb_12_O_18_] nanotube results in an unusual set of bands in the immediate vicinity of the Fermi level. Because such “in‐gap” states can, in principle, invert the customary ordering of band parities and thereby generate a topologically non‐trivial electronic phase, we evaluated the corresponding topological invariant. The band gap of the compound is identified as topologically trivial with Z_2_ index (0;000). Wannier states corresponding to the LUMO, constructed as linear combinations of three local Te 5*p*_σ_ orbitals, exhibit an antibonding Te‑centered 5*p*_σ∗_ configuration with sizable Sb‑ and O‑centered contributions (Figure S11).

### 2.5 Reaction Conditions and Alternative Products

Finally, we want to discuss the chemical question, why the compound **1** and especially the (SbOTe_3_)^3–^ anion forms in alkaline media. A mixture of solid KOH and water in the molar ratio *q*(K) = *n*(H_2_O):*n*(KOH) = 5.6, corresponding to a concentration of 10 mol L^−1^ (10 m), was found to be most suitable for the high yield synthesis of cetineite **1**. The starting materials are α‐Sb_2_O_3_ and elemental tellurium. A large fraction of the Sb_4_O_6_ molecules recrystallizes as [Sb_12_O_18_] nanotubes in the cetineite **1**, that is, with a strongly modified structural motive that seems to be templated by the interaction of the oxygen atoms with the hydrated K^+^ cations. A previous study revealed that antimony (III), at a considerably higher base concentration of *q*(K) = 1.0–1.8, can effectively reduce tellurium from +IV to negative oxidation states, resulting in its oxidation to antimony(V) [22]. That approach proved to be ineffective in this instance. Since a similar reaction of TeO_2_ with As_2_O_3_ in a KOH hydroflux with *q*(K) = 1.9 recently facilitated the formation of the anion (Te_3_)^2–^ [23], we added As_2_O_3_ to promote the reduction of Te to Te^−II^. Equation 2 shows that this unusual redox chemistry is favored by the high concentration of KOH in the reaction medium.

(2) $$\begin{array}{cc} & 7\;{\mathrm{Sb}}_{2}^{\mathrm{III}}O_{3}+6Te^{0}{+2\mathrm{As}}_{2}^{\mathrm{III}}O_{3}+18\mathrm{KOH}\to \\ & K_{6}\left[{\mathrm{Sb}}_{12}^{\mathrm{III}}O_{18}\right]{\left(Sb^{V}\mathrm{OT}{e^{-\mathrm{II}}}_{3}\right)}_{2}\cdot 5H_{2}O\left(1\right)+4K_{3}As^{V}O_{4}+4H_{2}O \end{array}$$

**FIGURE 6:**
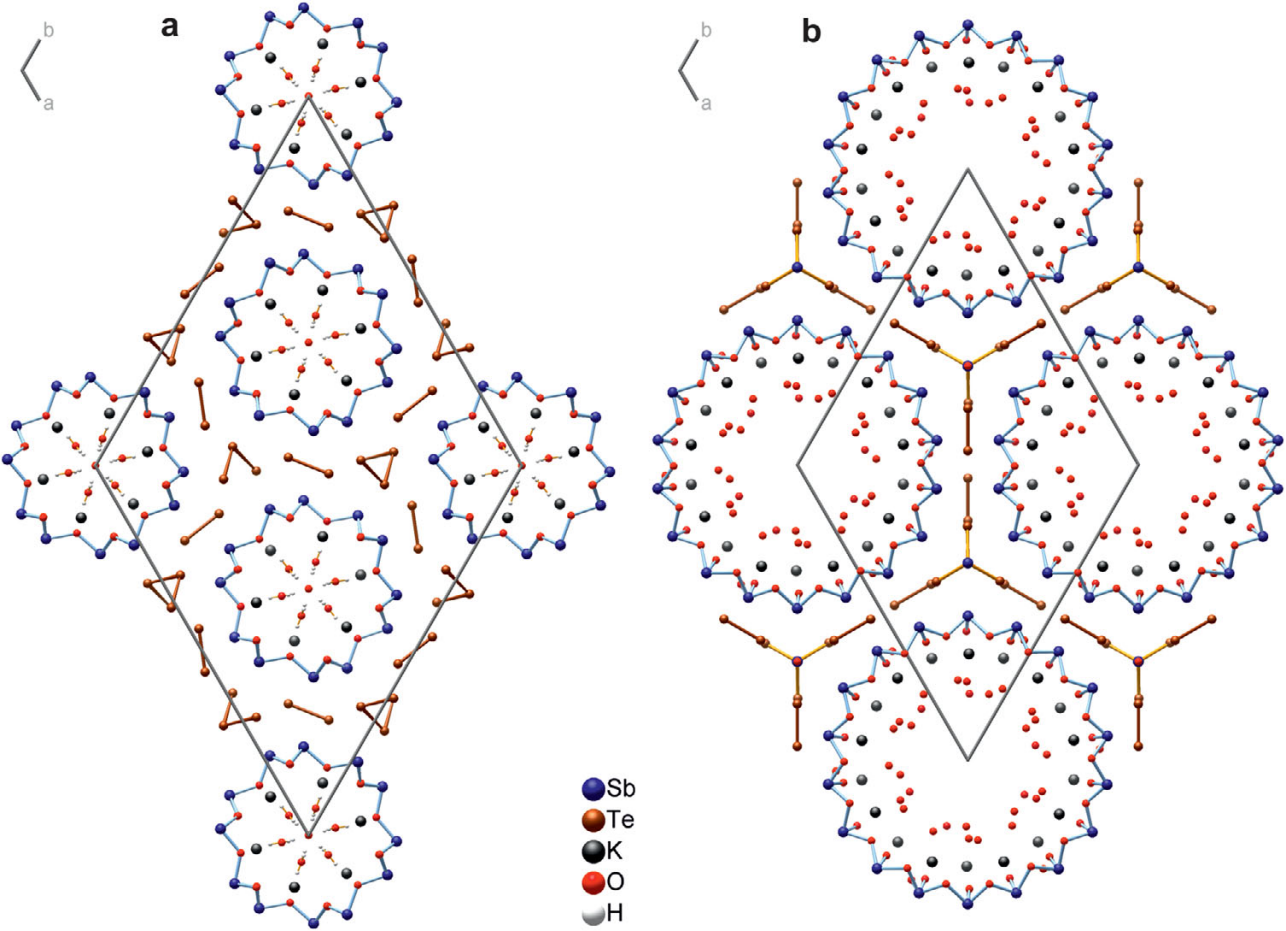
Crystal structures of (a) the tellurium‐rich cetineite **2** and (b) the wide‐diameter nanotube cetineite **3**.

In the course of finding the optimal reaction conditions for the formation of **1**, we tested different base concentrations *q*(K) as well as several starting materials (Table S1). Compounds with cetineite‐type structures crystallized only at moderate base concentrations, that is, in comparatively water‐rich systems with molar ratios *q*(K) = 5.6 or 11.1. When using either TeO_2_ instead of elemental tellurium and/or omitting As_2_O_3_, the tellurium‐rich cetineite K_6_[Sb_12_O_18_](Te_3_)_2_(Te_2_)_3_·5H_2_O (**2**; Figure S1a and Table S1) crystallized from a more diluted alkaline solution with *q*(K) = 11.1. This compound had been previously synthesized at 180°C from tellurium and antimony in an aqueous KOH solution with *q*(K) = 12, but using *n*‐propylamine as a templating agent [7]. In the structure of **2**, (Te_2_)^2−^ dumbbells compensate for the cation charge, while uncharged 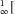 Te_3_] helices, known from the element structure, wind along the screw rotation axes in the rhombohedral structure (Figure 6a, Table S4 and S8). These results show that Sb_2_O_3_ is nevertheless sufficient to reduce tellurium(IV) even in alkaline solutions with comparatively low base concentrations (Equation 3). An alternative to the analogous reaction with elemental tellurium (Equation 4) is the disproportionation of tellurium in alkaline solution

(3) $$\begin{array}{cc} & {39\mathrm{Sb}}_{2}^{\mathrm{III}}O_{3}+24Te^{\mathrm{IV}}O_{2}+66\mathrm{KOH}+139H_{2}O\to \\ & 2K_{6}\left[{\mathrm{Sb}}_{12}^{\mathrm{III}}O_{18}\right]{\left({\mathrm{Te}}_{3}^{0}\right)}_{2}{\left({\mathrm{Te}}_{2}^{-I}\right)}_{3}\cdot 5H_{2}O\left(2\right)+54K\left[Sb^{V}{\left(\mathrm{OH}\right)}_{6}\right] \end{array}$$

(4) $$\begin{array}{cc} & {15\mathrm{Sb}}_{2}^{\mathrm{III}}O_{3}+24Te^{0}+18\mathrm{KOH}+19H_{2}O\to \\ & 2K_{6}\left[{\mathrm{Sb}}_{12}^{\mathrm{III}}O_{18}\right]{\left({\mathrm{Te}}_{3}^{0}\right)}_{2}{\left({\mathrm{Te}}_{2}^{-1}\right)}_{3}\cdot 5H_{2}O\left(2\right)+6K\left[Sb^{V}{\left(\mathrm{OH}\right)}_{6}\right] \end{array}$$

Comparing reactions (3) and (4) with reaction (2) shows that the As^III^_2_O_3‐_containing system benefits more from a high base concentration. This includes the hypothetical formation of cetineite **1** from an As^III^_2_O_3_‐free solution (Equation 5), which provides an explanation for our experimental findings

(5) $$\begin{array}{cc} & {9\mathrm{Sb}}_{2}^{\mathrm{III}}O_{3}+6Te^{0}+10\mathrm{KOH}+12H_{2}O\to \\ & K_{6}\left[{\mathrm{Sb}}_{12}^{\mathrm{III}}O_{18}\right]{\left(Sb^{V}{\mathrm{OTe}}_{3}^{-\mathrm{II}}\right)}_{2}\cdot 5H_{2}O\left(1\right)+4K\left[Sb^{V}{\left(\mathrm{OH}\right)}_{6}\right] \end{array}$$

At *q*(K) = 5.6 and also using either TeO_2_ instead of elemental tellurium and/or omitting As_2_O_3_, we obtained the new compound **K**_18_[Sb_36_O_54_](Sb**O**Te_3_)_2_(Te_2_)_6_·**22H_2_O** (**3**; Figures 6b, S1b, and S2b; and Tables S1, S3, and S4). It resembles **Rb**_18_[Sb_36_O_54_](SbTe_3_)_2_(Te_2_)_6_ [8] and contains [Sb_36_O_54_] nanotubes with a wide diameter of about 1.5 nm (based on O···O diagonal distances). In addition to the different alkaline metal cations and the finding of water molecules in the tubes, cetineite **3** appears to contain (SbOTe_3_)^3−^ anions in place of the (SbTe_3_)^3−^ anions in the rubidium compound. However, disorder impedes the unambiguous determination of the structure. The previously reported solvothermal syntheses of the rubidium compound started from mixtures of tellurium and antimony in an aqueous RbOH solution with *q*(Rb) = *n*(H_2_O): *n*(RbOH) = 12.5 and used *n*‐propylamine as a templating agent [8].

## 3 Conclusion

K_6_[Sb_12_O_18_](SbOTe_3_)_2_·5H_2_O (**1**) is a new member of the cetineite family. It includes the previously unknown oxidotritelluridoantimonate(V) anion (SbOTe_3_)^3−^. In this anion, antimony(V) is bonded directly to tellurium(−II), that is, two neighboring heavy elements with opposite maximum oxidation states share their valence electrons. As can be expected, the actual electron density distribution is less extreme. On the one hand, the alkaline medium used in hydrothermal synthesis promotes the formation of this anion. On the other hand, comparatively water‐rich systems with a concentration of 5 to 10 mol L^−1^ are required. Otherwise, different compounds, for example, **2** or **3**, which are also of the cetineite type, are formed. The main component of compound **1** is Sb_2_O_3_, which forms microporous, single‐walled [Sb_12_O_18_] nanotubes. Antimony(III) atoms form the outer and oxygen atoms the inner wall of the nanotube, making it radially dipolar, similar to a cylindrical capacitor. The spatial separation of the potassium cations inside the nanotubes and the (SbOTe_3_)^3−^ anions outside enhances this electrostatic effect. Substantial charge transfer from the (SbOTe_3_)^3−^ anions to the [Sb_12_O_18_] nanotubes causes heavy n‐doping of the latter. Consequently, the crystals of cetineite **1** are black and exhibit semiconducting behavior along their axis. This is despite the fact that the [Sb_12_O_18_] nanotubes have a wide intrinsic band gap of 3.6 eV, which corresponds to a colorless insulator.

## Conflicts of Interest

The authors declare no conflicts of interest.

## Supporting information



The Supporting Information includes experimental details and additional results of the synthesis and the crystal structure determinations, powder x‐ray diffraction data, SEM images, EDX analyses, thermal analysis, electrical resistivity measurement and DFT calculations. The authors have cited additional references within the Supporting Information [24, 25, 26, 27, 28, 29, 30, 31, 32, 33, 34, 35, 36, 37]. Further details of the crystal structure investigations may be obtained from the joint CCDC/FIZ Karlsruhe online deposition service: https://www.ccdc.cam.ac.uk/structures/ by quoting the deposition numbers CSD‐2517217, CSD‐2517227, CSD‐2517234, CSD‐2517238 for cetineite **1** (100 K, 298 K, 323 K, 343 K), and CSD‐2517121 for cetineite **3**. **Supporting File**: chem71106‐sup‐0001‐SuppMat.pdf

## Data Availability

The data that support the findings of this study are available from the corresponding author upon reasonable request.
