# Supplementary material for: Semiconducting Single‐Wall Antimony Oxide Nanotubes and Oxidotritelluridoantimonate(V) Anions
Source: Chemistry. 2026 May 11;32(31):e71106. doi: 10.1002/chem.71106 (PMC13485215; doi:10.1002/chem.71106)
Supplement: Supplementary file 1 [file CHEM-32-e71106-s001.pdf]

# Semiconducting Single-Wall Antimony Oxide Nanotubes and Oxidotritelluridoantimonate(V) Anions

Huanyu He, Oleg Janson, Vitaliy Romaka, and Michael Ruck

## Table of Contents

**1. Synthesis and Chemical Analysis .......... 2**

1.1. Synthesis of $K_6[Sb_{12}O_{18}](SbOTe_3)_2{\cdot}nH_2O$ (n = 5) (1) .......... 2

1.2. Synthesis of $K_6[Sb_{12}O_{18}](Te_3)_2(Te_2)_3{\cdot}5H_2O$ (2) .......... 2

1.3. Synthesis of $K_{18}[Sb_{36}O_{54}](SbOTe_3)_2(Te_2)_6{\cdot}22H_2O$ (3) .......... 3

**2. Scanning Electron Microscopy (SEM) and Energy Dispersive X-ray (EDX) Spectroscopy. 3**

**3. Powder X-ray Diffraction .......... 4**

**4. Crystal Structure Determination .......... 5**

**5. Thermal Analysis .......... 10**

**6. Electric Resistivity Measurement .......... 11**

**7. DFT calculations .......... 11**

## 1. Synthesis and Chemical Analysis

### 1.1. Synthesis of $K_6[Sb_{12}O_{18}](SbOTe_3)_2 \cdot nH_2O$ (n = 5) (1)

In a hydrothermal synthesis with a KOH concentration of 10 M ($q$(K) ≈ 5.6), long, metallic black, needle-shaped crystals were obtained. The reaction was carried out for 10 hours at 250 °C in the autoclave together with PTFE inlets preventing water loss. Subsequent to this, a cooling process at a rate of –1 K·min$^{-1}$ was initiated to reduce the temperature to ambient conditions. Using Te, $Sb_2O_3$ and $As_2O_3$ as starting materials, a single phase could be achieved and proved by chemical analysis using powder X-ray diffraction. Following the complete reaction, the product was filtered, extensively washed with water to eliminate any residual potassium hydroxide, and then dried under vacuum at room temperature. The compound **1** was obtained as a single phase with an approximate yield of 94%.

### 1.2. Synthesis of $K_6[Sb_{12}O_{18}](Te_3)_2(Te_2)_3 \cdot 5H_2O$ (2)

Metallic black, rod-shaped crystals were obtained under hydrothermal conditions in 5 M KOH solution ($q$(K) ≈ 11.1). (Figure S1a). The synthesis procedure and temperature program were the same as described above. Crystals of **2** were formed when $Sb_2O_3$ was reacted with elemental Te or $TeO_2$, omitting $As_2O_3$. When the synthesis was proceeded for longer time, e.g. reaching 48 hours, $K_{18}[Sb_{36}O_{54}](SbOTe_3)_2(Te_2)_6$ (**3**) crystallized.

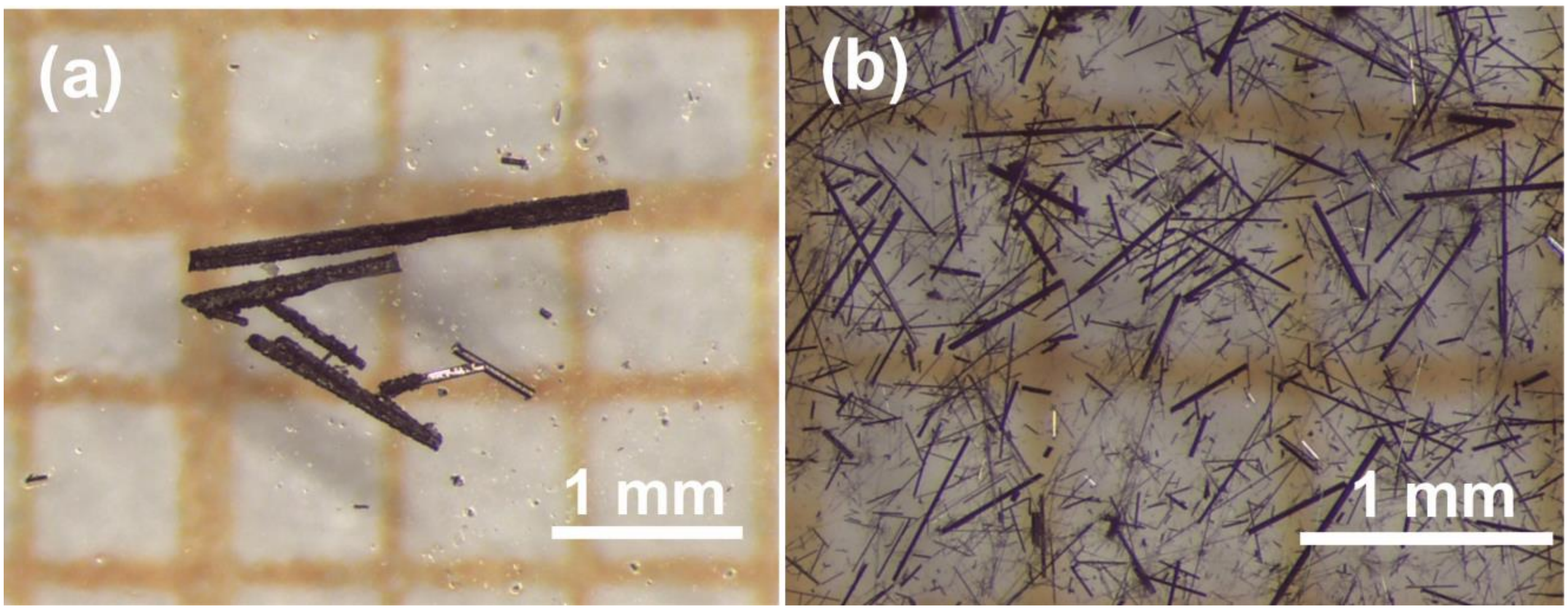


**Figure S1.** Photographs of crystals of (a) cetineite **2** and (b) cetineite **3**.

### 1.3. Synthesis of $K_{18}[Sb_{36}O_{54}](SbOTe_3)_2(Te_2)_6\cdot 22H_2O$ (3)

Thin, black, needle-shaped crystals of 3 (Figure S1b) were obtained under hydrothermal conditions in 10 M KOH solution ($q$(K) ≈ 5.6) using Te and $Sb_2O_3$ as starting materials. The synthesis procedure and temperature program were the same as described above.

**Table S1.** Product formation depending on the starting materials and the water-base-ratio.

| Starting Material | 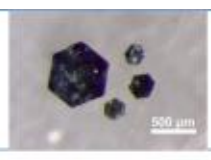 | | 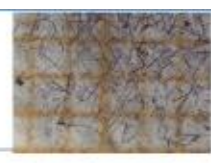 | 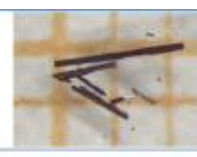 |
|---|---|---|---|---|
| $Te + Sb_2O_3$ | $K_2Sb_4O_6Te$ + $KSbO_5$ | | cetineite **3** | cetineite **2** + Te |
| $TeO_2 + Sb_2O_3$ | $K_2Sb_4O_6Te$ + $KSbO_5$ | | cetineite **3** + Te | cetineite **2** |
| | 1.0 1.3 1.8 | $q(K)=n(H_2O):n(KOH)$ | 5.6 | 11.1 |
| $Te + Sb_2O_3 + As_2O_3$ | $K_2Sb_4O_6Te$ | 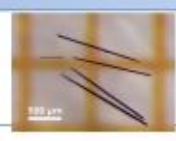 | cetineite **1** | cetineite **1** |
| $TeO_2 + Sb_2O_3 + As_2O_3$ | $K_2Sb_4O_6Te$ | | cetineite **3** | cetineite **2** |

cetineite **1:** $K_6(Sb_2O_3)_6[SbOTe_3]_2\cdot nH_2O$ $n \approx 5$

cetineite **2**: $K_6(H_2O)_6(Sb_2O_3)_6Te_{36}\cdot nH_2O$ Ref. K-SBC-1 (ICSD:413484)

cetineite **3:** $K_{18}(Sb_{36}O_{54})_6(SbTe_3)_2(Te_2)_6$ Ref. Rb-CTH-1 (ICSD:416106)

The synthesis of all related cetineites was carried out in a KOH (86 %, Fisher Scientific) hydrothermal environment. All the following materials were utilized directly upon purchase: Te (99.999 %, ChemPur), $Sb_2O_3$ (99.6 %, abcr), $TeO_2$ (99.9%, abcr) and $As_2O_3$ (99.996%, abcr). For the syntheses of cetineite **1** (0.5 mmol Te, 2.5 mmol $Sb_2O_3$, and 1 mmol $As_2O_3$), cetineite **2** (0.5 mmol Te and 2.5 mmol $Sb_2O_3$), and cetineite **3**, the respective precursor mixtures were individually dissolved or homogeneously suspended in 3 mL of deionized water, followed by the addition of the concentrated base as described above. In Table S1 regarding the formation of $K_2Sb_4O_6Te$ under high-concentration alkaline conditions, as mentioned in our previous work[27], further details on synthesis and properties will be published subsequently.

## 2. Scanning Electron Microscopy (SEM) and Energy Dispersive X-ray (EDX) Spectroscopy

Scanning electron microscopy (SEM) was performed using a SU8020 (Hitachi) with a triple detector system for secondary and low-energy backscattered electrons ($U_a$ = 5 kV; Figure S2). The composition of selected crystals was determined by semi-quantitative energy dispersive X-ray (EDX) analysis ($U_a$ = 15 kV; Table S2 and S3) using a Silicon Drift Detector (SDD) X-MaxN (Oxford Instruments). The data were processed (integration, pulse-pile up correction) applying the AZtec software package (Oxford Instruments, 2013).

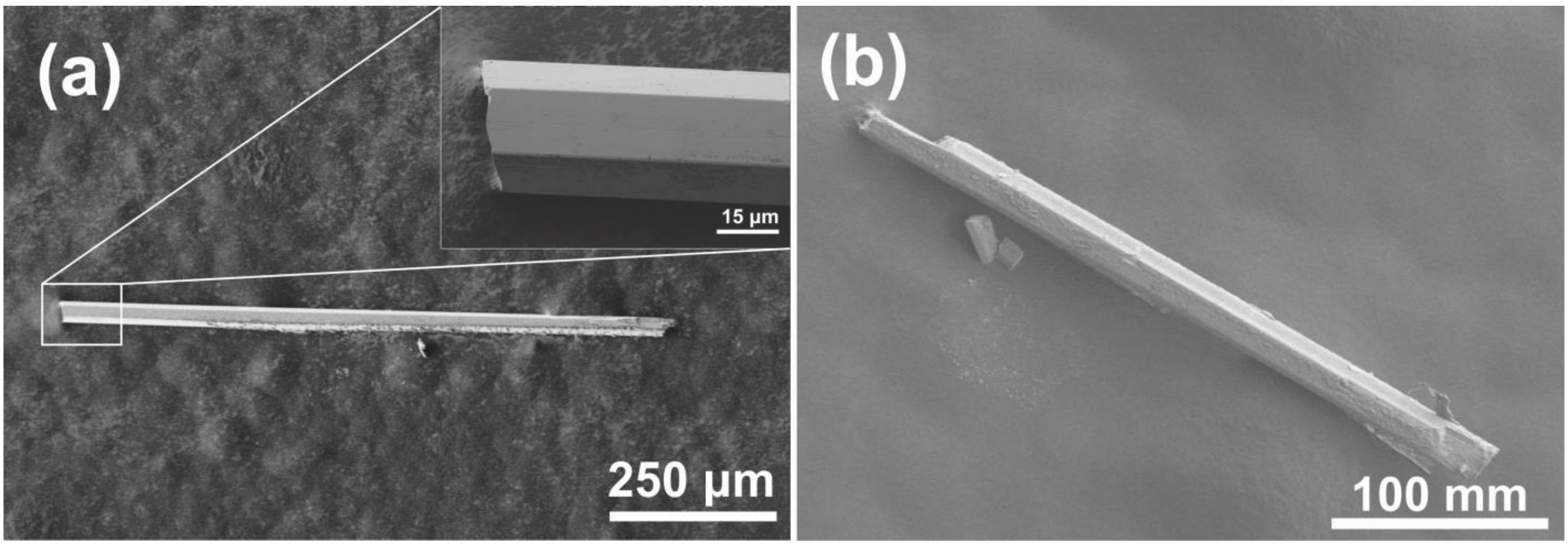


**Figure S2.** SEM images of a needle-like single-crystals of (a) cetineite **1** and (b) cetineite **3**.

**Table S2**. Results of the energy-dispersive X-ray spectroscopy (EDX) of $K_6(Sb_2O_3)_6[SbOTe_3]_2\cdot nH_2O$ ($n$≈5) (**1**). The standard deviations are given in brackets.

| | K | Sb | O | Te |
|---|---|---|---|---|
| at.-% | 14.5(1) | 28.1(1) | 46.(2) | 11.2(2) |
| molar ratio | 1.3(1) | 2.5(1) | 4.1(1) | 1.0(1) |
| theoretical ratio | 1.0 | 2.3 | 4.2 | 1.0 |

**Table S3**. Results of the energy-dispersive X-ray spectroscopy (EDX) of $K_{18}[Sb_{36}O_{54}](SbOTe_3)_2(Te_2)_6$ (**3**). The standard deviations are given in brackets.

| | K | Sb | O | Te |
|---|---|---|---|---|
| at.-% | 19(1) | 32(2) | 36(4) | 14(1) |
| molar ratio | 1.4(1) | 2.3(1) | 2.7(3) | 1.0(1) |
| theoretical ratio | 1.0 | 2.1 | 3.1 | 1.0 |

### 3. Powder X-ray Diffraction

X-ray powder diffraction patterns for phase identification were measured at room temperature on an Empyrean (Pananalytical) equipped with a curved Ge(111) monochromator using Cu-K$\alpha_1$ radiation ($\lambda$ = 154.056 pm). The intensities of the measured diffraction pattern of cetineite **1** (Figure S3) are strongly affected by the preferred orientation of the needle-shaped crystals. Grinding the sample only moderately improved the match with the calculated pattern.

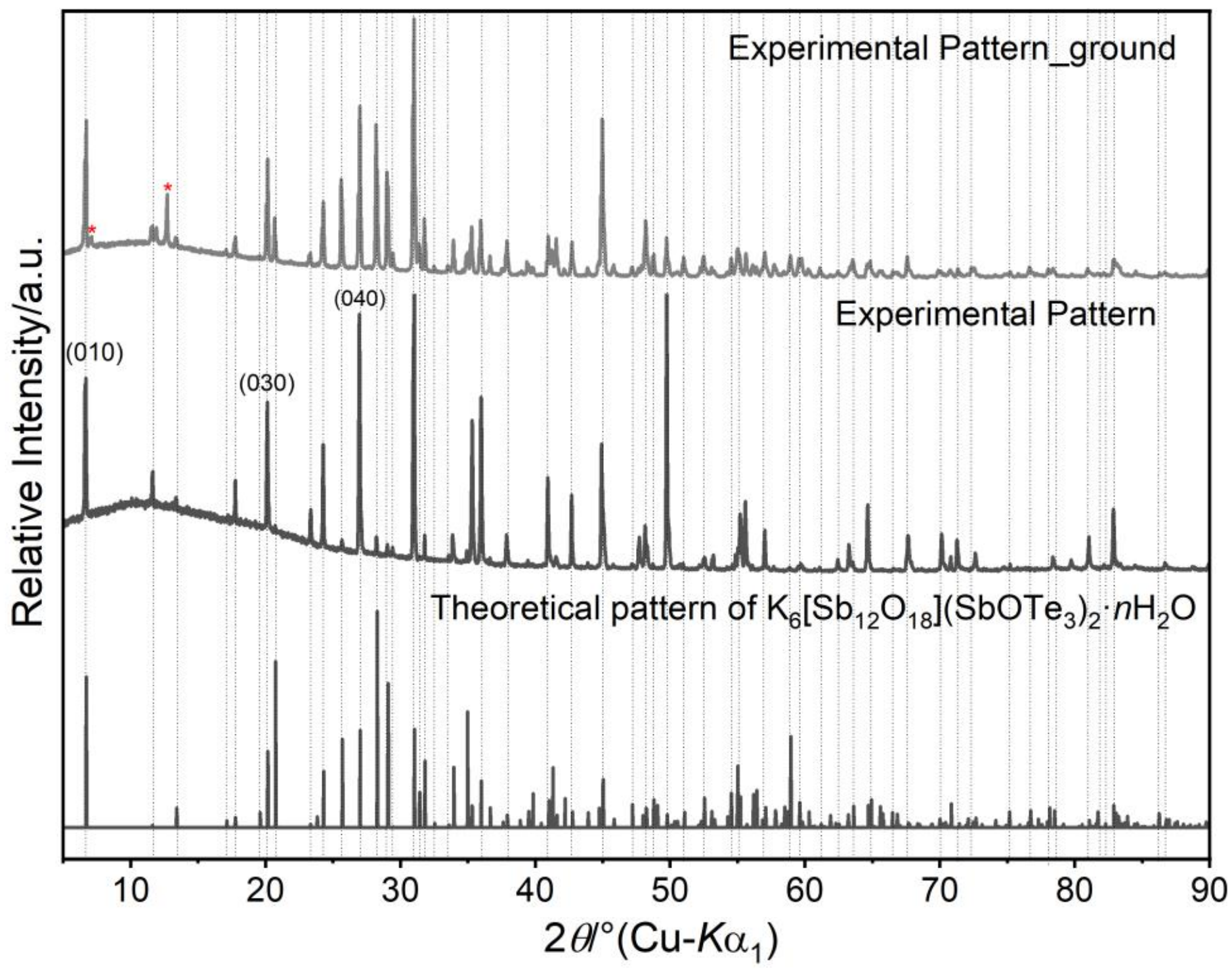


**Figure S3.** Powder X-ray diffractogram of the reaction product from cetineite **1** synthesis.

## 4. Crystal Structure Determination

Diffraction data of $K_6[Sb_{12}O_{18}](SbOTe_3)_2 \cdot 5H_2O$ (**1**) were collected at 100 K, 298 K, 323 K, and 343 K with a four-circle diffractometer Kappa Apex2 (Bruker) equipped with a CCD-detector using graphite-monochromated Mo-Kα radiation (Table S4). The raw data were corrected for background, Lorentz and polarization factors, and multi-scan absorption correction was applied. The structures were solved using ShelXT.[24] Structure refinement against $F^2$ with ShelXL[25] included anisotropic displacement parameters for all atoms except for the hydrogen atom. All graphical representations of the structure were developed with Diamond.[26] Table S4 to S7 contain crystal structure data.

The data set of **1** at 100 K was tested for merohedral twinning. The disorder of the $(SbOTe_3)^{3-}$ anions was not resolved upon reducing the space group symmetry to *P*3. Due to strong correlations the split position for Te could not be refined. Instead, *z*(Te) was optimized stepwise until a minimum of $wR_2$ was reached (Figure S4) and then fixed. Anisotropic displacement parameters were refined for all non-hydrogen atoms, incl. Te atoms. Nonetheless, there is a comparatively high residual electron density close to the Te position. The occupancies of the water molecules (atoms O4 and O5) were refined and then fixed close to these values. The positions of the hydrogen atoms were refined by constraining the O–H distance to 90(1) pm and assigning the 1.2-fold of the isotropic displacement parameter of the bonded O atom.

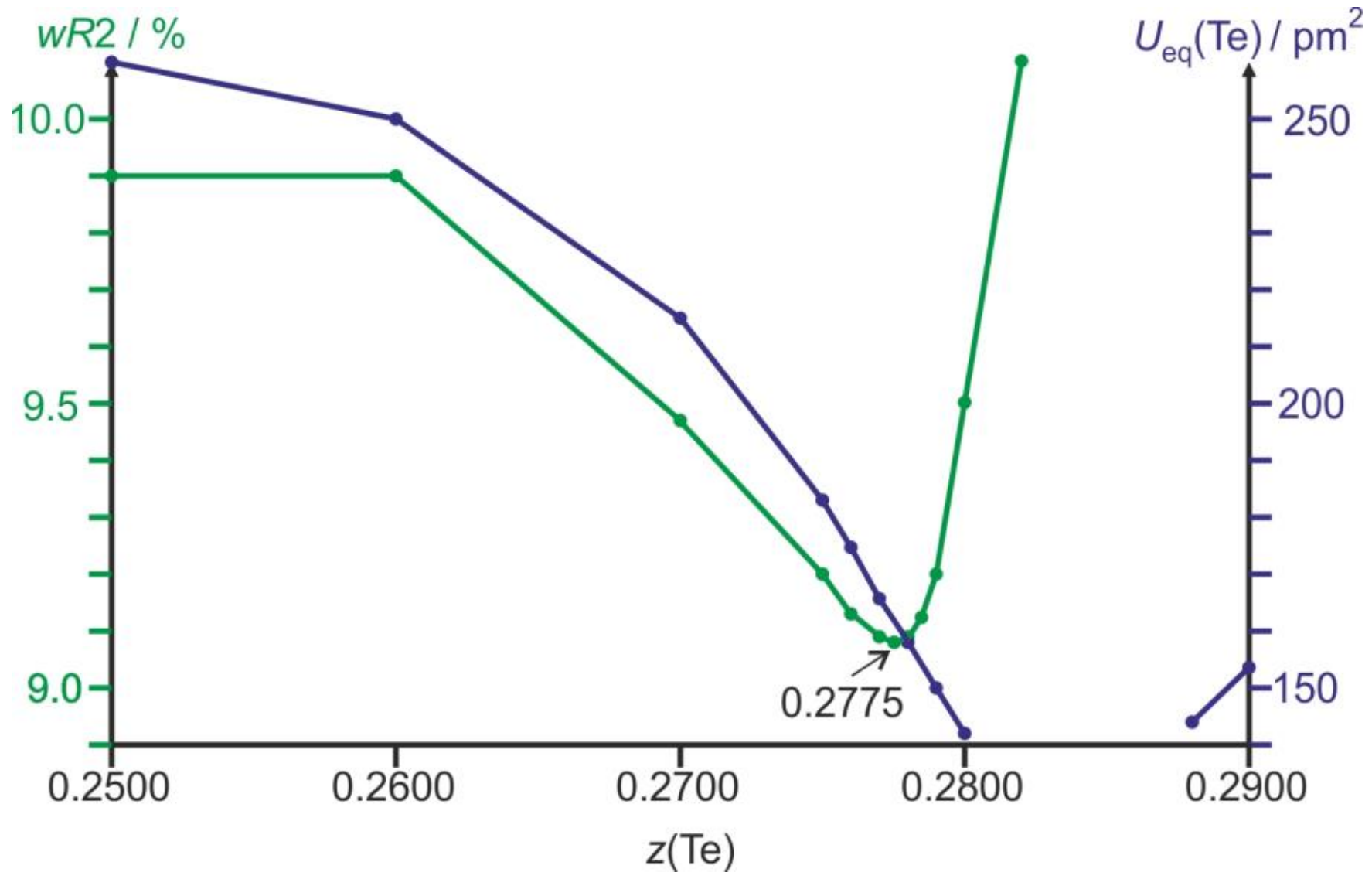


**Figure S4.** Dependence of *wR2* and of the equivalent isotropic displacement parameter of the Te atom in cetineite **1** on the *z* coordinate of the Te atom.

Diffraction data of $K_6[Sb_{12}O_{18}](Te_3)_2(Te_2)_3 \cdot 5H_2O$ (**2**) were collected at 100 K and 298 K with a four-circle diffractometer Kappa Apex2 (Bruker) equipped with a CCD-detector using graphite-monochromated Mo-Kα radiation (Table S4). The data processing was conducted in the manner delineated above. Structure refinement included anisotropic displacement parameters for all atoms except for oxygen atoms belonging to water molecules with low occupancies and the hydrogen atom. Table S4 and S8 contain crystal structure data.

Diffraction data of $K_{18}[Sb_{36}O_{54}](SbOTe_3)_2(Te_2)_6 \cdot 22H_2O$ (**3**) were collected at 298 K with a four-circle diffractometer Kappa Apex2 (Bruker) equipped with a CCD-detector using graphite-monochromated Mo-Kα radiation (Table S4). The data processing was conducted in the manner delineated above. Structure refinement included anisotropic displacement parameters for all atoms except for the atoms belonging to the water molecules. Table S4 and S9 contain crystal structure data.

**Table S4.** Crystal data and structure refinement for $K_6[Sb_{12}O_{18}](SbOTe_3)_2{\cdot}5H_2O$ (**1**), $K_6[Sb_{12}O_{18}](Te_3)_2(Te_2)_3{\cdot}5H_2O$ (**2**) and $K_{18}[Sb_{36}O_{54}](SbOTe_3)_2(Te_2)_6{\cdot}22H_2O$ (**3**).

| | | | |
|---|---|---|---|
| Empirical formula | $K_6[Sb_{12}O_{18}](SbOTe_3)_2{\cdot}5H_2O$ | $K_6[Sb_{12}O_{18}]Te_{12}{\cdot}5H_2O$ | $K_{18}[Sb_{36}O_{54}](SbOTe_3)_2Te_{12}{\cdot}22H_2O$ |
| Structure type | cetineite | cetineite | cetineite |
| Formula weight | 3114.78 | 3604.88 | 8919.45 |
| Temperature | 100(2) K | 100(2) K | 298(2) |
| Crystal system, $Z$ | hexagonal, 1 | trigonal, 3 | hexagonal, 1 |
| Space group (number) | $P6_3/m$ (176) | $R$ -3 (148) | $P6_3/m$ (176) |
| $a$ / pm | 1523.92(5) | 2722.69(6) | 2177.81(3) |
| $c$ / pm | 562.21(2) | 566.82(2) | 976.05(1) |
| $V$ / ($10^6$ pm$^3$) | 1130.72(8) | 3638.9(2) | 4009.1(1) |
| $\rho_{calc}$ / (g cm$^{-3}$) | 4.574 | 4.935 | 3.694 |
| Diffractometer | Bruker Kappa APEX II CCD, graphite (002) monochromator | | |
| $\mu$ / mm$^{-1}$ | 12.6 | 14.2 | 10.0 |
| Crystal size / mm$^3$ | 0.100 × 0.040 × 0.014 | 0.110 × 0.030 × 0.020 | 0.330 × 0.070 × 0.030 |
| Crystal color | black | black | black |
| Crystal shape | needle | needle | needle |
| Radiation | Mo$K_\alpha$ ($\lambda$ = 71.073 pm) | | |
| $2\theta_{max}$ / ° | 90.4 | 67.7 | 68.0 |
| Index range | $-30 \le h,k \le 30$<br>$-11 \le l \le 11$ | $-41 \le h,k \le 41$<br>$-8 \le l \le 8$ | $-34 \le h,k \le 34$<br>$-14 \le l \le 14$ |
| Reflections measured | 74437 | 21610 | 154926 |
| Unique reflections | 3360 | 2583 | 5510 |
| $R_{int}$ | 0.068 | 0.061 | 0.061 |
| $R_\sigma$ | 0.024 | 0.038 | 0.025 |
| Parameters | 62 | 97 | 132 |
| Restraints | 2 | 1 | 1 |
| Extinction coefficient | $4(1){\cdot}10^{-4}$ | — | — |
| Goodness-of-fit on $F^2$ | 1.031 | 1.026 | 1.102 |
| $R_1$ [$F_o > 4\sigma(F_o)$] | 0.035 [2792] | 0.028 [2267] | 0.052 [4577] |
| $R_1$ [all $F_o$] | 0.045 | 0.036 | 0.068 |
| w$R_2$ [all $F_o$] | 0.091 | 0.055 | 0.114 |
| Residual electron density / (e $10^{-6}$ pm$^{-3}$) | –5.67 e (44 pm from Sb3)<br>to 7.10 (81 pm from Te) | –1.39 to 1.40 | –3.84 (0.43 A from Sb4)<br>to 3.08 (0.25 A from Te3b) |

**Table S5.** Lattice parameters for $K_6[Sb_{12}O_{18}](SbOTe_3)_2{\cdot}5H_2O$ (**1**) at various temperatures.

| $T$ / K | 100(2) | 298 K | 323 K | 343 K |
|---|---|---|---|---|
| $a$ / pm | 1523.92(5) | 1525.65(2) | 1526.69(3) | 1526.06(3) |
| $c$ / pm | 562.21(2) | 562.41(1) | 562.74(1) | 562.26(1) |
| $V$ / ($10^6$ pm$^3$) | 1130.72(8) | 1133.69(3) | 1135.90(5) | 1133.99(5) |

**Table S6.** Wyckoff positions, coordinates, occupancies, and tensor coefficients of the anisotropic displacement parameters (/ pm$^2$) for the atoms in $K_6[Sb_{12}O_{18}](SbOTe_3)_2{\cdot}5H_2O$ (**1**) at 100 K, 298 K, 323 K, and 343 K (space group *P*6$_3$/*m*). The equivalent isotropic displacement parameter $U_{eq}$ (/ pm$^2$) is defined as one third of the orthogonalized displacement tensor.

| Atom | W.p. | *x* | *y* | *z* | *sof* | $U_{11}$ | $U_{22}$ | $U_{33}$ | $U_{12}$ | $U_{13}$ | $U_{23}$ | $U_{eq}$ |
|---|---|---|---|---|---|---|---|---|---|---|---|---|
| Te | 12*i* | 0.15613(3) | 0.52576(3) | 0.27748 | ½ | 113(1) | 119(1) | 231(3) | 39.8(9) | –19(1) | –1(1) | 162(1) |
| | | 0.15662(6) | 0.52696(6) | | | 276(3) | 303(3) | 446(6) | 98(3) | –19(5) | 19(5) | 362(2) |
| | | 0.15665(6) | 0.52696(6) | | | 299(3) | 327(3) | 464(5) | 103(3) | –22(5) | 19(5) | 387(2) |
| | | 0.15676(7) | 0.52685(7) | | | 323(3) | 352(4) | 506(6) | 113(3) | –24(6) | 23(6) | 419(2) |
| Sb1 | 6*h* | 0.38287(2) | 0.36363(2) | 1/4 | 1 | 125(1) | 110.9(9) | 57.0(8) | 68.0(8) | 0 | 0 | 93.6(5) |
| | | 0.38219(4) | 0.36306(4) | | | 223(2) | 211(2) | 147(2) | 118(2) | | | 189(2) |
| | | 0.38195(4) | 0.36285(4) | | | 242(2) | 226(2) | 155(2) | 129(2) | | | 203(1) |
| | | 0.38171(4) | 0.36267(4) | | | 263(3) | 248(3) | 171(3) | 141(2) | | | 221(1) |
| Sb2 | 6*h* | 0.42068(2) | 0.15042(2) | 1/4 | 1 | 126(1) | 102.6(9) | 57.7(8) | 53.6(8) | 0 | 0 | 96.8(5) |
| | | 0.42005(4) | 0.15005(4) | | | 224(3) | 211(2) | 153(2) | 105(2) | | | 197(2) |
| | | 0.41987(4) | 0.15010(4) | | | 239(2) | 224(2) | 161(2) | 111(2) | | | 210(1) |
| | | 0.41973(4) | 0.15015(4) | | | 260(3) | 243(3) | 180(3) | 122(2) | | | 229(2) |
| Sb3 | 4*f* | 1/3 | 2/3 | 0.4414(2) | 1/2 | 121(2) | 121(2) | 213(3) | 60.3(7) | 0 | 0 | 151(1) |
| | | | | 0.4419(3) | | 298(4) | 298(4) | 347(8) | 149(2) | | | 314(3) |
| | | | | 0.4422(3) | | 320(4) | 320(4) | 360(7) | 160(2) | | | 334(3) |
| | | | | 0.4422(3) | | 343(5) | 343(5) | 384(9) | 172(2) | | | 357(4) |
| K | 6*h* | 0.09314(8) | 0.25706(8) | 1/4 | 1 | 145(3) | 208(4) | 154(3) | 99(3) | 0 | 0 | 164(2) |
| | | 0.0933(2) | 0.2575(2) | | | 292(9) | 400(10) | 279(9) | 194(8) | | | 315(4) |
| | | 0.0932(2) | 0.2573(2) | | | 314(9) | 430(10) | 290(9) | 206(8) | | | 338(4) |
| | | 0.0929(2) | 0.2568(2) | | | 340(10) | 470(10) | 310(10) | 231(9) | | | 367(5) |
| O1 | 12*i* | 0.2786(2) | 0.3352(2) | 0.0011(4) | 1 | 151(8) | 150(8) | 53(6) | 91(7) | –19(6) | 0(6) | 111(3) |
| | | 0.2787(3) | 0.3348(3) | 0.0017(7) | | 260(20) | 290(20) | 140(20) | 150(20) | –20(10) | 10(10) | 226(8) |
| | | 0.2783(3) | 0.3346(3) | 0.0014(7) | | 300(20) | 310(20) | 150(20) | 180(20) | –40(10) | 10(10) | 243(7) |
| | | 0.2785(3) | 0.3346(3) | 0.0019(8) | | 310(20) | 340(20) | 170(20) | 180(20) | –20(20) | 20(20) | 261(8) |
| O2 | 6*h* | 0.3356(3) | 0.2143(3) | 1/4 | 1 | 190(10) | 120(10) | 120(10) | 90(10) | 0 | 0 | 138(5) |
| | | 0.3353(5) | 0.2140(5) | | | 300(30) | 220(30) | 270(30) | 150(20) | | | 260(10) |
| | | 0.3352(5) | 0.2140(4) | | | 350(30) | 230(30) | 270(30) | 160(20) | | | 270(10) |
| | | 0.3347(5) | 0.2139(5) | | | 340(30) | 280(30) | 290(30) | 200(20) | | | 290(10) |
| O3 | 2*d* | 1/3 | 2/3 | 3/4 | 1 | 57(9) | 57(9) | 60(10) | 29(4) | 0 | 0 | 58(6) |
| | | | | | | 160(20) | 160(20) | 250(50) | 80(10) | | | 190(20) |
| | | | | | | 190(20) | 190(20) | 290(50) | 90(10) | | | 220(20) |
| | | | | | | 220(30) | 220(30) | 310(50) | 110(10) | | | 250(20) |
| O4 | 6*h* | 0.1355(5) | 0.0870(6) | 1/4 | 2/3 | 150(20) | 270(30) | 510(50) | 70(20) | 0 | 0 | 320(20) |
| | | 0.134(1) | 0.087(1) | | | 270(60) | 460(70) | 1000(100) | 120(50) | | | 590(40) |
| | | 0.134(1) | 0.087(1) | | 0.32(2) | 350(70) | 600(100) | 1000(100) | 230(60) | | | 640(60) |
| | | 0.133(1) | 0.087(1) | | 0.31(2) | 360(80) | 600(100) | 1100(200) | 200(70) | | | 680(70) |
| H4 | 12*i* | 0.099(7) | 0.065(8) | 0.12(1) | 2/3 | | | | | | | 1.2·$U_{eq}$(O4) |
| | | 0.096(8) | 0.07(1) | 0.12(1) | 2/3 | | | | | | | |
| | | 0.097(8) | 0.06(1) | 0.12(1) | 0.65(4) | | | | | | | |
| | | 0.097(7) | 0.06(1) | 0.12(1) | 0.62(4) | | | | | | | |
| O5 | 2*b* | 0 | 0 | 0 | 1/2 | 170(30) | 170(30) | 320(70) | 80(10) | 0 | 0 | 220(20) |
| | | | | | 1/2 | 360(70) | 360(70) | 300(100) | 180(30) | | | 340(50) |
| | | | | | 0.49(4) | 380(90) | 380(90) | 400(100) | 190(40) | | | 390(70) |
| | | | | | 0.49(6) | 500(100) | 500(100) | 500(200) | 240(60) | | | 500(100) |
| H5 | 12*i* | 0.06(2) | 0.06(2) | 0.02(10) | 1/6 | | | | | | | 1.2·$U_{eq}$(O5) |
| | | 0.063(6) | 0.03(6) | 0.06(4) | 1/6 | | | | | | | |
| | | 0.065(5) | 0.03(6) | 0.05(4) | 0.16(1) | | | | | | | |
| | | 0.063(4) | 0.03(5) | 0.06(3) | 0.16(2) | | | | | | | |

**Table S7.** Selected interatomic distances (/ pm) in cetineite **1** at 100(1) K.

| Atom pair | | Distance | | Atom pair | | Distance | |
|---|---|---|---|---|---|---|---|
| Sb1– | O1 | 1.996(2) | 2× | K– | O1 | 2.828(2) | 4× |
| | O2 | 2.014(3) | | | O4 | 2.968(8) | |
| Sb2– | O1 | 1.968(2) | 2× | | O4 | 2.985(8) | |
| | O2 | 1.973(4) | | | O2 | 3.001(1) | 2× |
| Sb3– | O3 | 1.7351(9) | | | O4 | 3.246(4) | 2× |
| | Te | 2.7601(5) | 3× | O4– | H4 | 0.90(1) | 2× |
| | | | | O5– | H5 | 0.90(1) | 2× |

**Table S8.** Coordinates, occupancies and coefficients of the displacement tensor $U_{ij}$ (/ pm$^2$) of the atoms in $K_6[Sb_{12}O_{18}](Te_3)_2(Te_2)_3 \cdot 5H_2O$ (**2**) at 100(2) K. $U_{eq}$ (/ pm$^2$) is defined as one third of the trace of the orthogonalized tensor $U_{ij}$.

| Atom | *x* | *y* | *z* | *sof* | $U_{11}$ | $U_{22}$ | $U_{33}$ | $U_{23}$ | $U_{13}$ | $U_{12}$ | $U_{eq}$; $U_{iso}$ |
|---|---|---|---|---|---|---|---|---|---|---|---|
| Sb1 | 0.55894(2) | 0.09449(2) | 0.06934(5) | 1 | 52(1) | 70(1) | 53(1) | 6.3(9) | 5.1(9) | 33(1) | 57.3(6) |
| Sb2 | 0.45183(2) | 0.13531(2) | 0.09383(5) | 1 | 65(1) | 53(1) | 58(1) | –3.2(9) | –1.3(9) | 34(1) | 56.8(6) |
| Te1 | 0.37024(2) | –0.01428(2) | 0.90232(5) | 1 | 95(1) | 63(1) | 72(1) | 0.3(9) | –0.3(10) | 42(1) | 75.6(7) |
| Te2 | 0.46352(2) | 0.02204(2) | 0.50175(5) | 1 | 76(1) | 121(1) | 116(1) | 52(1) | 36(1) | 49(1) | 104.7(7) |
| K1 | 0.70404(5) | 0.22957(5) | 0.0859(2) | 1 | 97(5) | 225(6) | 132(5) | 6(4) | 15(4) | –12(4) | 193(2) |
| O1 | 0.6142(1) | 0.1420(1) | –0.1786(5) | 1 | 90(10) | 80(10) | 60(10) | 1(10) | 10(10) | 4(10) | 77(6) |
| O2 | 0.4738(1) | 0.1924(1) | 0.3484(5) | 1 | 110(10) | 70(10) | 60(10) | –10(10) | 8(10) | 4(10) | 81(6) |
| O3 | 0.5318(1) | 0.1504(1) | 0.0800(5) | 1 | 80(10) | 110(10) | 100(10) | 8(10) | –5(10) | 70(10) | 86(6) |
| O4 | 0.6135(3) | 0.2606(3) | 0.089(2) | 0.792(2) | 310(40) | 180(30) | 1400(90) | 10(40) | –50(50) | 80(30) | 650(30) |
| O5 | 2/3 | 1/3 | 0.219(11) | 0.0352(8) | | | | | | | 200 |
| O6 | 2/3 | 1/3 | –0.049(11) | 0.0352(8) | | | | | | | 200 |
| H1 | 0.589(5) | 0.231(5) | 0.09(2) | 0.652(2) | | | | | | | 200 |
| H2 | 0.627(5) | 0.278(5) | –0.04(2) | 0.652(2) | | | | | | | 200 |
| H3 | 0.710(6) | 0.346(7) | 0.23(3) | 0.421(3) | | | | | | | 200 |

**Table S9.** Coordinates and coefficients of the displacement tensor $U_{ij}$ (/ $pm^2$) of the atoms in $K_{18}[Sb_{36}O_{54}](SbOTe_3)_2(Te_2)_6 \cdot 22H_2O$ (**3**) at 298(2) K. $U_{eq}$ (/ $pm^2$) is defined as one third of the trace of the orthogonalized tensor $U_{ij}$.

| Atom | *x* | *y* | *z* | $U_{11}$ | $U_{22}$ | $U_{33}$ | $U_{23}$ | $U_{13}$ | $U_{12}$ | $U_{eq}$; $U_{iso}$ |
|---|---|---|---|---|---|---|---|---|---|---|
| Sb1 | 0.48693(3) | 0.24315(6) | 0.07214(4) | 362(2) | 270(4) | 123(2) | 0(40) | –9(2) | 228(4) | 221(1) |
| Sb2 | 0.36823(5) | 0.44982(5) | 0.07449(9) | 274(4) | 236(4) | 111(4) | –32(3) | –33(3) | 118(3) | 212(2) |
| Sb3 | 0.08270(4) | 0.44962(5) | 0.07511(8) | 106(3) | 298(4) | 9.(3) | 15(3) | –1(3) | 86(3) | 172(2) |
| Sb4 | 2/3 | 1/3 | 0.6188(3) | 960(10) | 390(6) | 390(6) | 456(12) | 0 | 0 | 412(5) |
| O1 | 0.4193(6) | 0.1456(5) | –0.0070(8) | 400(30) | 210(30) | 130(20) | –110(30) | –30(30) | 180(30) | 234(12) |
| O2 | 0.4359(4) | 0.2222(7) | 1/4 | 400(30) | 210(30) | 130(20) | -110(30) | –30(30) | 180(30) | 234(12) |
| O3 | 0.4191(6) | 0.2729(5) | 0.0156(7) | 350(30) | 350(30) | 50(20) | 60(30) | 0(30) | 210(30) | 232(13) |
| O4 | 0.3602(4) | 0.3655(4) | –0.002(1) | 250(30) | 330(30) | 168(19) | –10(40) | 0(40) | 220(20) | 215(10) |
| O5 | 0.3267(8) | 0.4026(8) | 1/4 | 250(30) | 330(30) | 168(19) | –10(40) | 0(40) | 220(20) | 215(10) |
| O6 | 0.0771(8) | 0.3997(9) | 1/4 | 350(30) | 350(30) | 50(20) | 60(30) | 0(30) | 210(30) | 232(13) |
| O7 | 2/3 | 1/3 | 0.480(1) | 530(40) | 530(40) | 430(70) | 0 | 0 | 270(20) | 500(30) |
| K1 | 0.3445(5) | 0.0675(4) | 1/4 | 1040(60) | 550(40) | 320(30) | 0 | 0 | 520(40) | 590(20) |
| K2 | 0.3440(3) | 0.2775(3) | 1/4 | 420(30) | 480(30) | 290(20) | 0 | 0 | 260(20) | 375(12) |
| K3 | 0.1829(4) | 0.3595(3) | 1/4 | 400(30) | 790(30) | 780(30) | 0 | 0 | 350(30) | 637(11) |
| Te1 | 0.5231(1) | 0.04655(4) | 1/4 | 389(8) | 275(3) | 365(4) | 0 | 0 | 124(8) | 362(2) |
| Te2 | 0.5947(1) | 0.18979(5) | 1/4 | 271(8) | 291(4) | 1193(11) | 0 | 0 | 121(8) | 593(3) |
| Te3a | 0.6032(2) | 0.3943(2) | 3/4 | 399(9) | 726(15) | 701(10) | 0 | 0 | 427(8) | 544(5) |
| Te3b | 0.5411(3) | 0.2793(4) | 3/4 | 399(9) | 726(15) | 701(10) | 0 | 0 | 427(8) | 544(5) |
| Te3c | 0.5688(8) | 0.337(1) | 3/4 | 399(9) | 726(15) | 701(10) | 0 | 0 | 427(8) | 544(5) |
| Ow1 | 0.222(2) | 0.241(2) | 0.022(4) | | | | | | | 1260(13) |
| Ow2 | 0.266(3) | 0.136(6) | 0.101(7) | | | | | | | 1260(13) |
| Ow3 | 0.106(2) | 0.271(2) | 0.015(5) | | | | | | | 1260(13) |
| Ow4 | 0.173(4) | 0.223(4) | 1/4 | | | | | | | 1260(13) |
| Ow5 | 0.224(7) | 0.11(2) | 1/4 | | | | | | | 1260(13) |
| Ow6 | 0.37(2) | 0.41(2) | 1/4 | | | | | | | 1260(13) |

### 5. Thermal Analysis

A sample of cetineite **1** was heated to 1200 °C at a rate of 5 K $min^{-1}$ in an alumina crucible under streaming argon inside a STA/PT1600 (Linseis); see Figure S5. The chemical composition of the residual after the TG experiment was analyzed with EDX spectroscopy (Table S10).

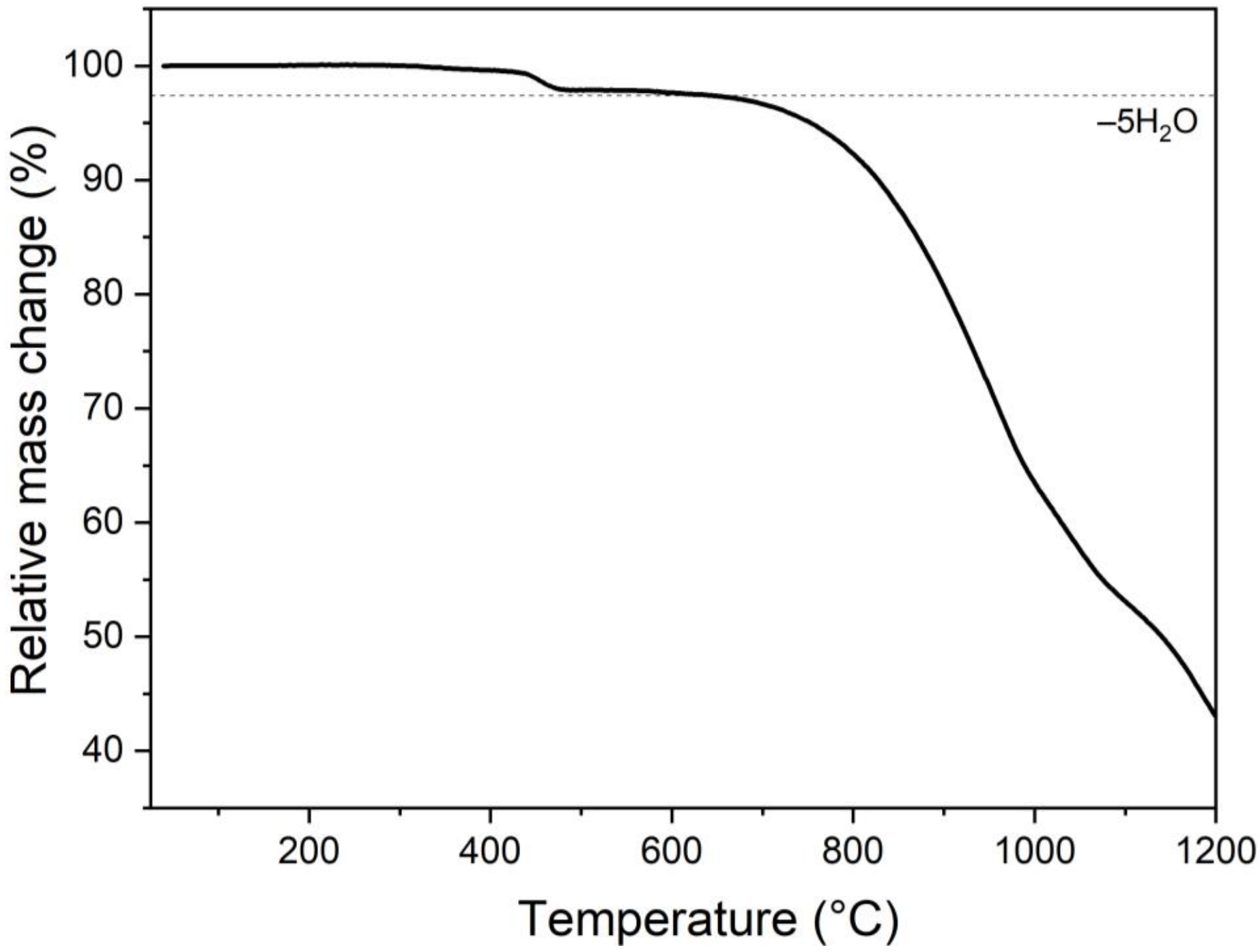


**Figure S5.** Thermogravimetry (TG) of the cetineite **1**.

**Table S10**. Results of the energy-dispersive X-ray spectroscopy (EDX) of cetineite **1** after thermogravimetry (TG) measurement.

| | K | Sb | O |
|---|---|---|---|
| at.-% | 11(3) | 18(8) | 70(10) |
| molar ratio | 0.6(2) | 1.0(4) | 3.7(5) |

### 6. Electric Resistivity Measurement

A direct current resistance (DCR) setup for a cryogen-free measurement system (CFMS; Cryogenic) was employed to measure the temperature-dependent resistivity (*T* = 2 to 300 K) of a single crystal of cetineite **1**. The four-probe technique was applied along the needle axis of the crystal using Ag paste in combination with Au wire (50 μm) as contacts (Figure S6).

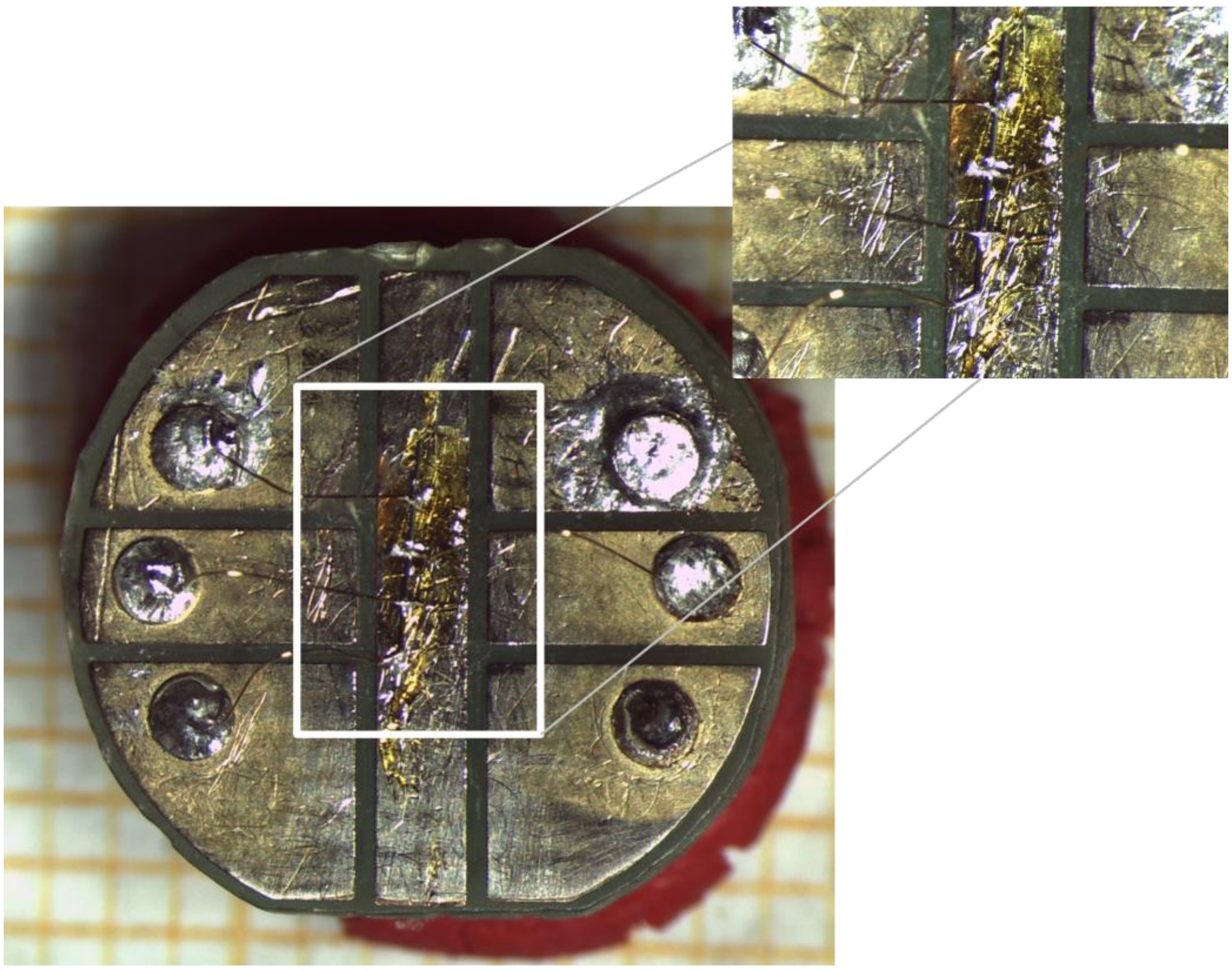

**Figure S6.** Set up for electric resistivity measurement along the needle axis of a crystal of cetineite (1)

### 7. DFT calculations

The DFT calculations were carried out using the Vienna Ab initio Simulation Package VASP v.5.4.4[28] with PAW-type potentials[29] and the Perdew-Burke-Enzerhoff (PBE) exchange-correlation functional in the generalized gradient approximation (GGA)[30] with the Monkhorst-Pack mesh resulted in 666 irreducible k-points[31]. The energy cutoff for the plane-wave basis set of 450 eV was selected based on the plane-wave cutoff of the corresponding elements, and the muffin-tin radii were automatically determined at the initial stage of calculations. The following valence electron configurations of the PAW potentials were used: Te (5$s^2$ 5$p^4$), Sb (5$s^2$ 5$p^3$), O (2$s^2$ 2$p^4$), K (3$s^2$ 3$p^6$ 4$s^1$), and H (1$s^1$). The final total energy calculations with and without spin-orbit coupling were performed using the completely relaxed crystal structures (atomic positions and lattice parameters) achieved by minimizing forces, stress tensor components, and total energies.
The Bader charge density analysis was performed with the Bader v1.05 code[32] and the population analysis with Lobster v5.1.1[33]. For plotting crystal structures, electron localization function, and charge density, the VESTA program[34] was used.
The nature of the band gap has been analyzed using GGA calculations performed with the full-potential local-orbital code FPLO version 22 [35] with the Wannier module [36]. The crystal structure of the dehydrated cetineite was optimized and additionally symmetrized to comply with the space group *P*-3 (147). For both scalar-and full-relativistic calculations, a k-mesh of 8 × 8 × 18 points has been employed. The topological analysis was performed using the parity algorithm [37] implemented in FPLO.

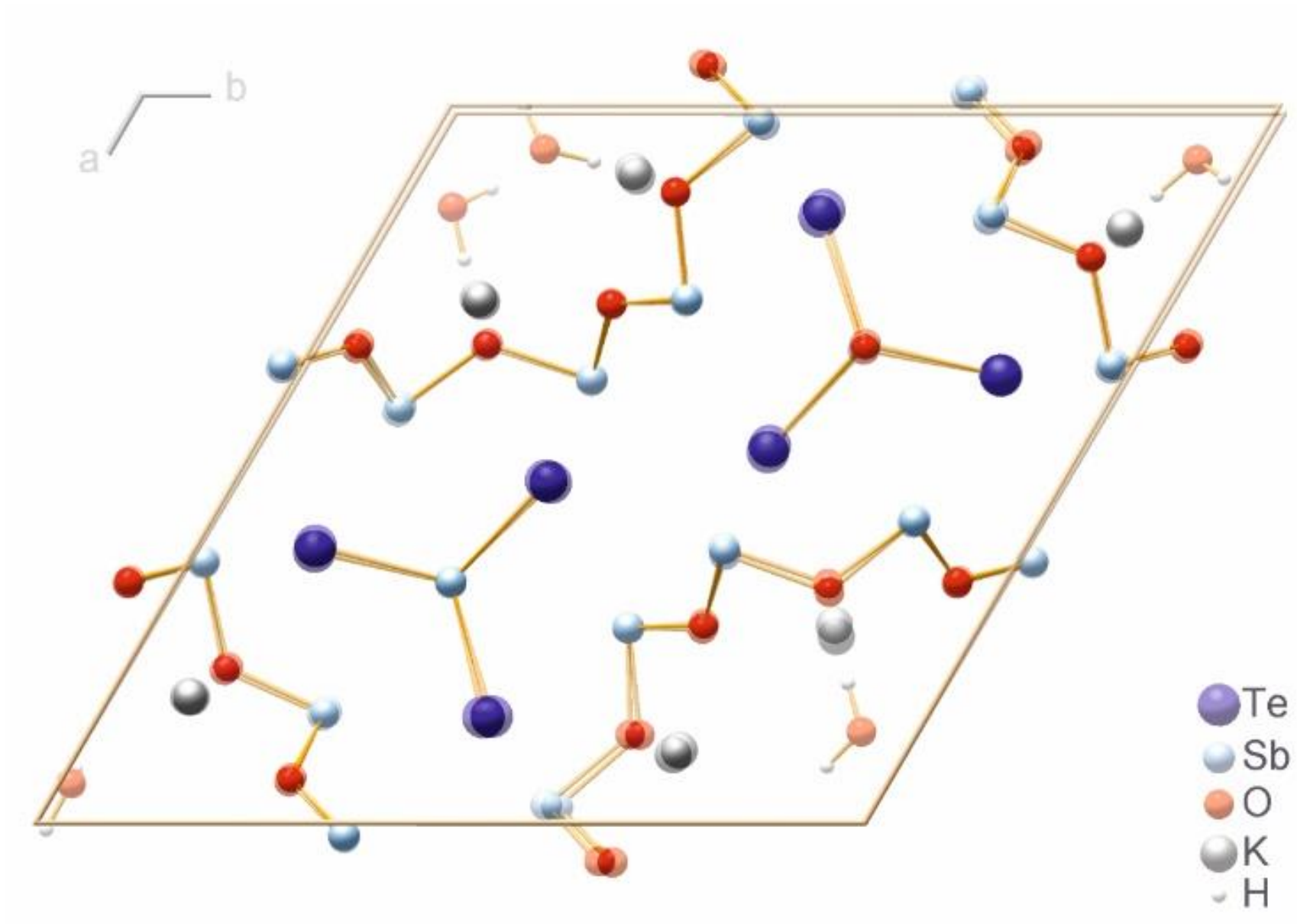


**Figure S7.** DFT-optimized unit cell of the water containing cetineite **1** superimposed with the optimized unit cell of its anhydrous form **1a**.

**Table S11.** Interatomic distances, Integrated Crystal Orbital Bond Index (ICOBI), Integrated Crystal Orbital Hamilton Population (ICOHP) at the Fermi level ($E_F$) of atom pairs in the DFT-optimized hydrated cetineite **1** and the dehydrated cetineite **1a**.

| | Cetineite hydrated | | | Cetineite dehydrated | | |
|---|---|---|---|---|---|---|
| | Distance (pm) | ICOBI | -ICOHP (eV) | Distance (pm) | ICOBI | -ICOHP (eV) |
| Sb3-O3 | 187.5 | 1.028 | 6.486 | 187.5 | 1.033 | 6.480 |
| Sb3-Te | 274.6 | 0.813 | 3.771 | 275.6 | 0.808 | 3.688 |
| Te-K | 356.9 | 0.084 | 0.548 | 354.8 | 0.086 | 0.467 |
| K-O2 | 288.1 | 0.026 | 0.180 | 296.2 | 0.030 | 0.160 |
| K-O1 | 283.9 | 0.039 | 0.263 | 279.9 | 0.037 | 0.215 |
| Sb2-O2 | 202.4 | 0.700 | 4.635 | 200.7 | 0.736 | 4.804 |
| Sb2-O1 | 202.2 | 0.700 | 4.672 | 202.2 | 0.694 | 4.599 |
| Sb1/Sb2-O1 | 200.7 (Sb1) | 0.730 | 4.863 | 204.9 (Sb2) | 0.632 | 4.269 |
| K-Ow | 315.3 | 0.029 | 0.187 | - | - | |
| Hw-Ow | 101.5 | 0.670 | 7.045 | - | - | |

**Table S12.** Bader, Mulliken, and Löwdin atomic charges (minimum and maximum) for the DFT-optimized crystal structures of hydrated hydrated cetineite **1** and dehydrated cetineite **1a**.

| Atom | Cetineite hydrated | | | Cetineite dehydrated | | |
|---|---|---|---|---|---|---|
| | Bader | Mulliken | Löwdin | Bader | Mulliken | Löwdin |
| Te | –0.565…–0.580 | –0.49…–0.51 | –0.42…–0.45 | –0.584…–0.586 | –0.53 | –0.46 |
| Sb1 | 1.607…1.588 | 1.33…1.37 | 1.19…1.22 | 1.602…1.601 | 1.36 | 1.22 |
| Sb2 | 1.704…1.694 | 1.46…1.47 | 1.32…1.33 | 1.694…1.686 | 1.46 | 1.33 |
| Sb3 | 1.128…1.116 | 0.92…0.93 | 0.92…0.93 | 1.115…1.115 | 0.94 | 0.94 |
| K | 0.857…0.850 | 0.83…0.86 | 0.70…0.71 | 0.849…0.849 | 0.83 | 0.71 |
| O1 | –1.185…–1.191 | –1.03…–1.04 | –0.92…–0.93 | –1.192…–1.197 | –1.05 | –0.94 |
| O2 | –1.162…–1.199 | –0.98…–1.01 | –0.87…–0.90 | –1.182…–1.188 | –1.03 | –0.92 |
| O3 | –1.075…–1.080 | –0.96…–0.97 | –0.92 | –1.061…–1.061 | –0.97 | –0.93 |
| Ow | –1.286…–1.328 | –0.92…–0.98 | –0.74…–0.80 | - | - | - |
| Hw | 0.649…0.619 | 0.40…0.46 | 0.33…0.37 | - | - | - |

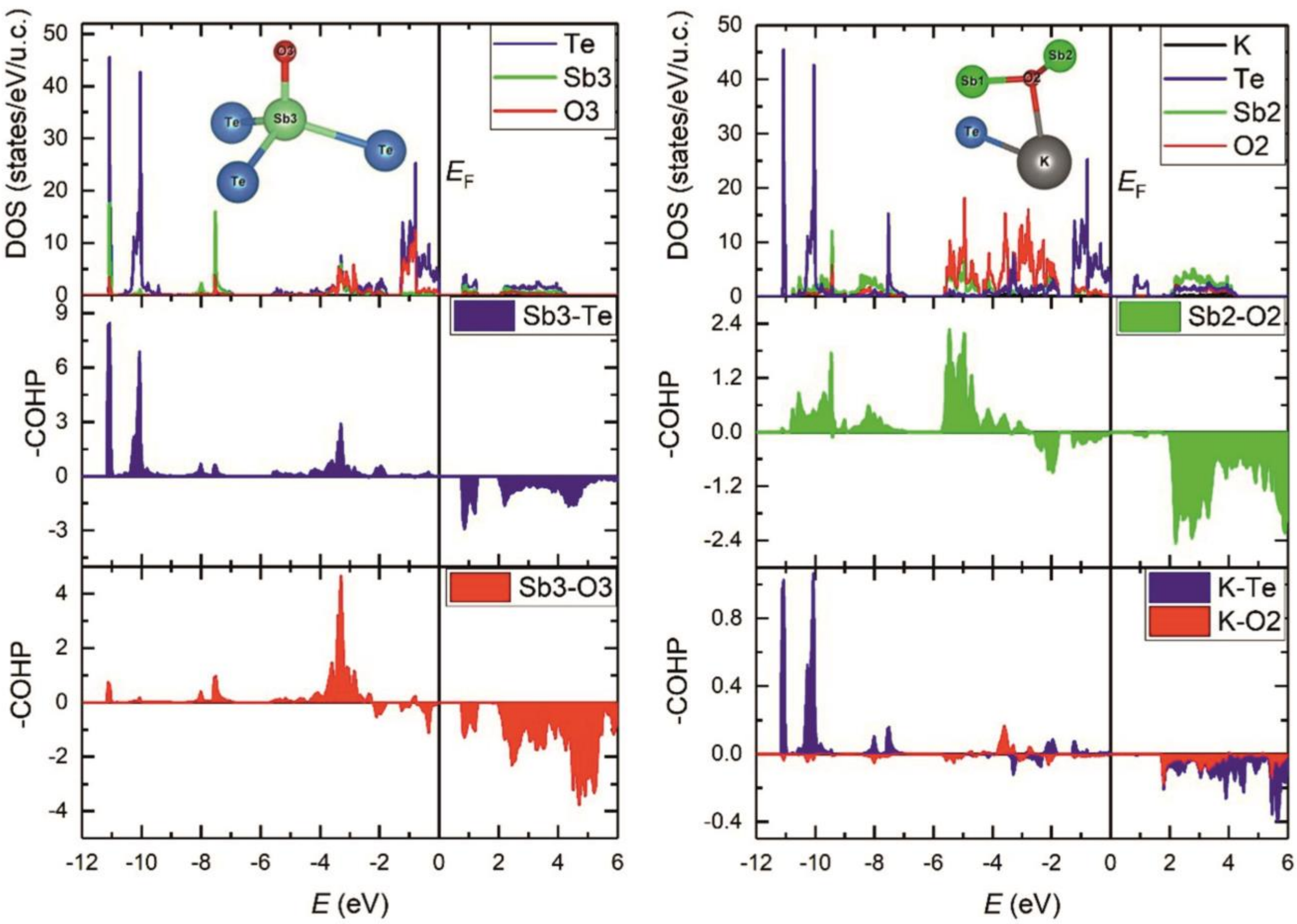


**Figure S8.** Cetineite **1:** (a) Distribution of the density of states (DOS) of Te, Sb3 and O3 atoms in comparison to the COHP of the Sb3-O3 and Sb3-Te atomic pairs. (b) DOS of K, Te, Sb2, and O2 atoms with COHP for Sb2-O2, K-Te, and K-O2 pairs.

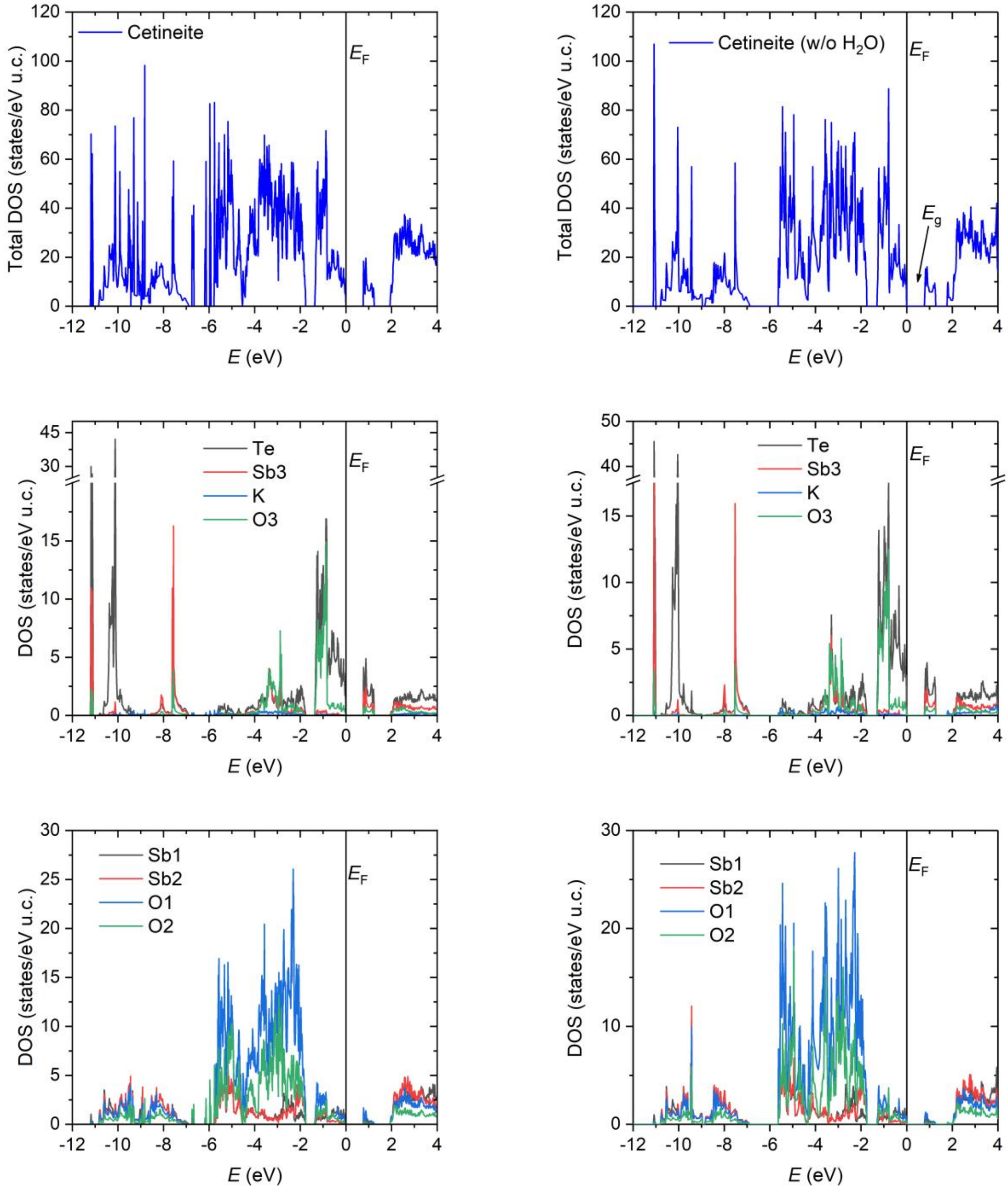


**Figure S9**. Distributions of the total and projected density of states in the DFT-optimized hydrated cetineite **1** and the dehydrated cetineite **1a**.

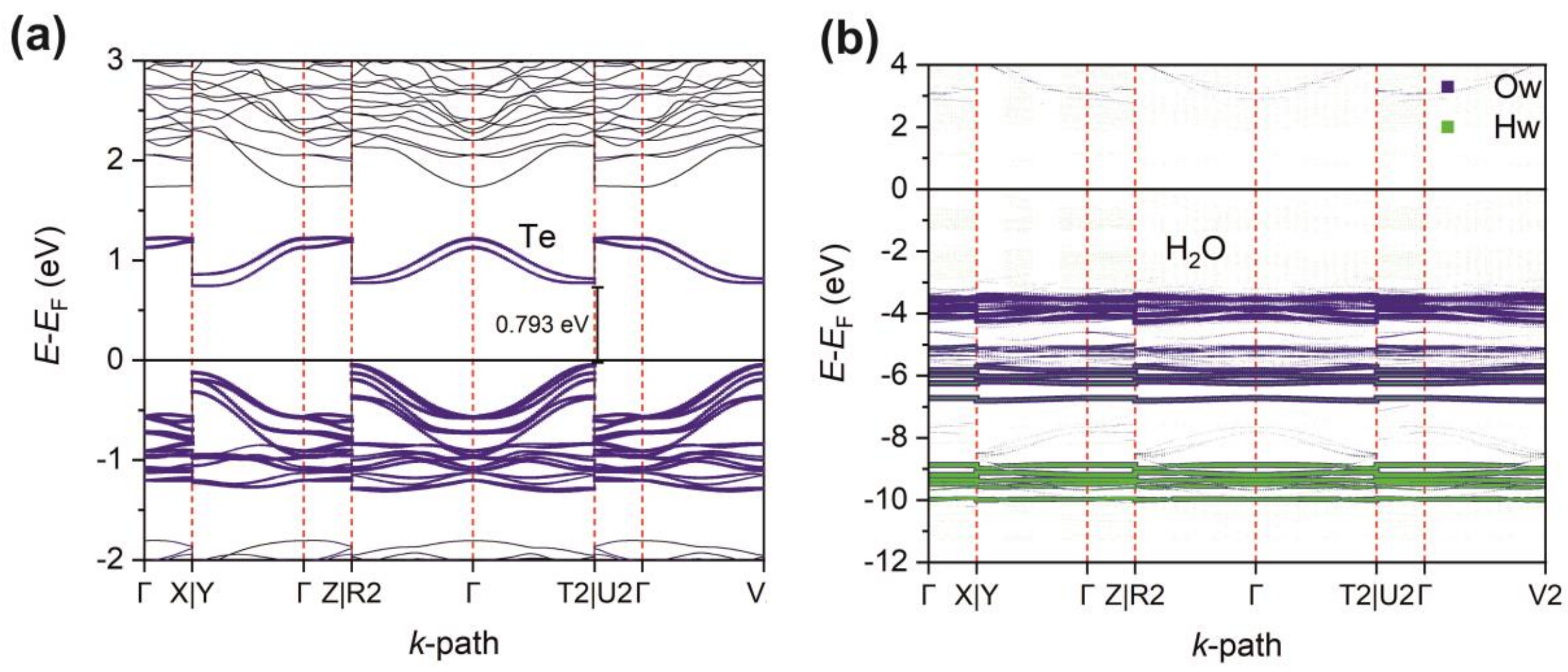


**Figure S10.** (a) Electronic band structure of the optimized dehydrated cetineite with an indirect band gap of 0.793 eV. (b). The impact of the water of crystallization on the band structure.

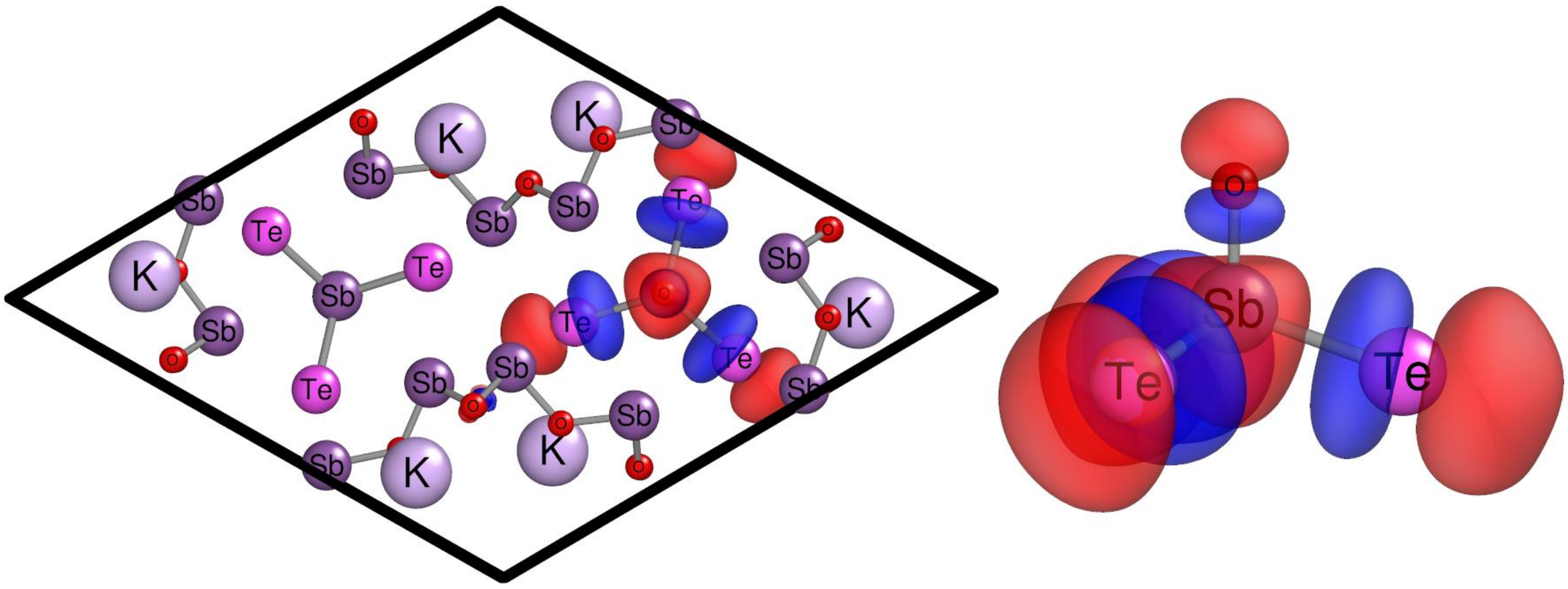


**Figure S11.** One of the two equivalent Wannier functions comprising the LUMO (a linear combination of three Te $5p_{\sigma*}$ orbitals): top (left panel) and side (right panel) views.
